# Straightforward Synthesis, Spectroscopic Characterizations, Molecular Docking, Molecular Dynamics Simulations, and Comprehensive DFT Calculations of Novel Tetrazole 1,5 and 2,5‐Disubstituted Tetrazole Scaffolds

**DOI:** 10.1002/open.70268

**Published:** 2026-08-05

**Authors:** Riham Sghyar, Ahmed Chelouan, Brahim El Ibrahimi, Olivier Blacque, Basavarajaiah Suliphuldevara Mathada, Prashantha Karunakar, Oussama Moussaoui, Mouad Lahyaoui, Joel T. Mague, Ali Aghmiz, Manal El‐Gendy, Mohamed Hefnawy, Nada Kheira Sebbar

**Affiliations:** ^1^ Applied Chemistry, Microbiology and Biotechnologies Department of Chemistry Faculty of Sciences University Abdelmalek Essaadi Tetouan Morocco; ^2^ Laboratory of Applied Organic Chemistry Faculty of Science and Techniques Sidi Mohamed Ben Abdellah University Fez Morocco; ^3^ Department of Applied Chemistry Faculty of Applied Sciences Ibnou Zohr University Aït Melloul Morocco; ^4^ Department of Chemistry University of Zurich Zurich Switzerland; ^5^ Department of Chemistry Dayananda Sagar Academy of Technology & Management (DSATM) Bangalore Karnataka India; ^6^ Department of Biotechnology Dayananda Sagar College of Engineering (Affiliated to Visvesvaraya Technological University Belagavi) Bangalore Karnataka India; ^7^ Laboratory of Organic Synthesis Extraction and Valorization‐Department of Chemistry Faculty of Sciences Ain Chock Hassan II University of Casablanca Casablanca Morocco; ^8^ Department of Chemistry Tulane University New Orleans Louisiana USA; ^9^ Department of Pharmaceutical Chemistry College of Pharmacy King Saud University Riyadh Saudi Arabia; ^10^ Laboratory of Heterocyclic Organic Chemistry Drug Science Research Center Pharmacochemistry Competence Center Faculty of Sciences Mohammed V University in Rabat Rabat Morocco

**Keywords:** alkylation, crystal structure, DFT calculation, docking studies, *N*,*N*‐diethylethylamine hydrochloride, tetrazoles

## Abstract

New 1,5‐ and 2,5‐disubstituted tetrazole derivatives were successfully synthesized under phase‐transfer catalysis conditions in good to excellent yields (21%–97%). The structures of the obtained compounds were confirmed by ^1^H and ^13^C NMR spectroscopy, as well as single‐crystal X‐ray diffraction analysis. Density functional theory calculations performed at the B3LYP/DNP 3.5 level showed that compound **F** possesses the smallest HOMO–LUMO energy gap (3.390 eV), suggesting enhanced chemical reactivity. Monte Carlo/SAA simulations demonstrated strong adsorption tendencies on metallic surfaces, particularly for compound **1j** on the Fe(110) surface, with an adsorption energy of −229.944 kcal mol^−1^. Molecular docking investigations against Abl kinase targets demonstrated favorable binding affinities, with docking scores reaching −11.7 and −8.3 kcal mol^−1^ for the most active derivatives. In addition, 100 ns molecular dynamics simulations confirmed the stability of the 2HZI–**3d** and 4TWP–**2j** complexes throughout the simulation period. These findings highlight the potential of the synthesized tetrazole derivatives as promising candidates for further biological and corrosion‐related applications.

## Graphical abstract

A series of novel 1,5‐ and 2,5‐disubstituted tetrazole heterocyclic scaffolds was synthesized and characterized by single‐crystal X‐ray diffraction. Their electronic properties were investigated using density functional theory calculations, while molecular docking and molecular dynamics simulations revealed promising biological potential and favorable protein–ligand interactions.

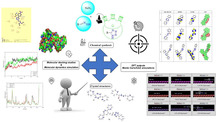

## 1 Introduction

**SCHEME 1:**
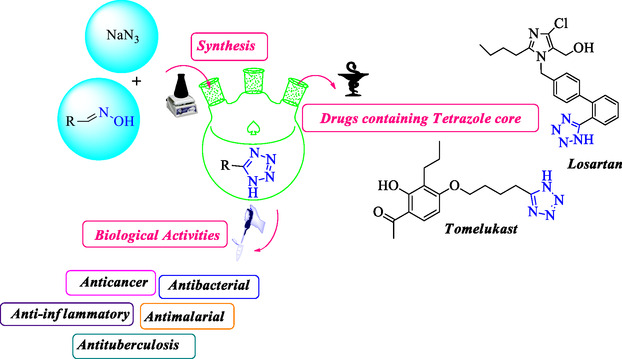
Illustration depicting the synthesis and biological applications of tetrazole, as well as the structure of some drugs that contain it.

The heterocyclic rings in tetrazoles play a significant role in their biological activities during drug discovery [1, 2, 3]. The pharmaceutical industry widely utilizes tetrazoles due to their nitrogen‐labile nature, planar geometry, low cytotoxicity, acidity, and lipophilicity. As a result, tetrazoles are particularly useful for the development of more bioactive compounds. For example, tetrazole‐family drugs, such as Losartan, a non‐peptide angiotensin II antagonist, have demonstrated excellent antihypertensive efficacy compared to carboxylic acid analogs [4, 5, 6, 7]. Another compound where the tetrazole moiety is significant is tomelukast, a leukotriene receptor antagonist, which demonstrated antiasthmatic activity 30 times higher when carboxylic acid groups were substituted with a 5‐substituted tetrazole moiety [8, 9, 10]. Additionally, 1,5‐ and 2,5‐disubstituted tetrazoles exhibit various biologically active properties, including antibacterial [11], antimalarial [12], anti‐inflammatory [13], anticancer [14], and antituberculosis activities [15, 16] Scheme 1.

Many research publications have reported novel methods for preparing tetrazoles, reflecting the growing importance of tetrazole groups across various fields, including coordination chemistry [17, 18], materials science [19, 20], and medicinal chemistry [21, 22]. Cycloaddition reactions are often employed to produce 5‐substituted 1H‐tetrazoles from nitrile and oxime substrates [23, 24, 25, 26, 27]. Various approaches have been documented in the literature for synthesizing 1,5‐ and 2,5‐disubstituted tetrazoles, with N‐alkylation being the most commonly utilized [26, 27, 28, 29, 30]. The reaction conditions influence the proportion of isomers generated. For instance, increasing the temperature generally promotes the formation of 1,5‐isomers. Additionally, the type of alkylating agent and the characteristics of the substituent at position 5, including steric hindrance [31, 32, 33] and electron‐withdrawing effects, affect the yield of 2,5‐isomers. Specifically, bulky substituents tend to steer alkylation towards the production of the 2,5‐isomer. The goal of this work is to synthesize and characterize new highly functionalized tetrazole compounds. Using phase transfer catalysis (PTC) in basic media at room temperature, we alkylated 5‐substituted tetrazoles with 2‐chloro‐*N*,*N*‐diethylamine hydrochloride and 4‐(3‐chloropropyl)−3,6‐dipyridazine. By spectroscopic analysis of ^1^H and ^13^C NMR spectra, the obtained products were fully characterized, while the structures of compounds **F**, **G,** and **1j** were unambiguously confirmed by single‐crystal X‐ray diffraction. To complement the experimental work, density functional theory (DFT) calculations at the B3LYP/DNP 3.5 level were performed to optimize geometries, predict spectral data, and generate Z‐matrix coordinates for all synthesized compounds **F**, **G**, and **1j**, as well as for Monte Carlo/SAA simulations. Finally, molecular docking and long‐term molecular dynamics (MD) simulations were performed to investigate the interactions and stability of selected compounds **F**, **G,** and **1j**.

## 2 Results and Discussion

### 2.1 Synthesis of 1,5‐ and 2,5‐Disubstituted Alkyl Tetrazole Derivatives

**SCHEME 2:**
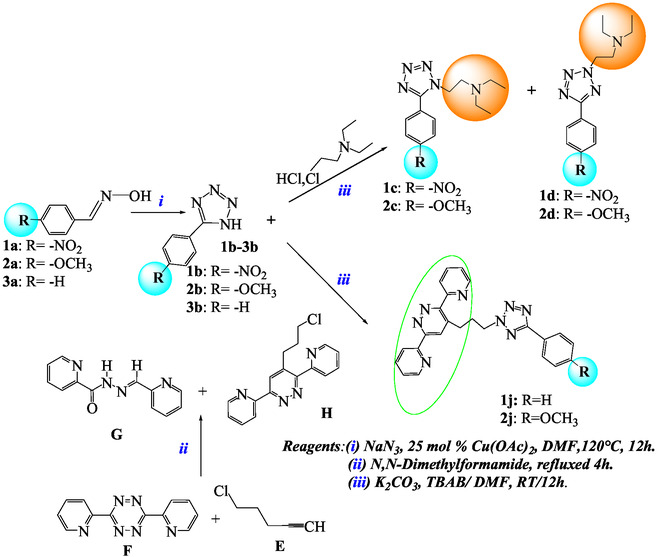
General synthetic route for 1,5‐ and 2,5‐substituted alkyl tetrazoles.

To synthesize new disubstituted tetrazole derivatives [**(1c‐2c/1d‐2d)‐(1j‐2j)**], we adopted the alkylation reaction between a variety of 5‐substituted tetrazoles and alkylating reagents such as 2‐chloro‐*N,N*‐diethylethylamine hydrochloride, 1‐(2‐chloroethyl) piperidine hydrochloride, and 4‐(3‐chloropropyl)−3,6‐di(pyridin‐2‐yl)pyridazine in the presence of a phase transfer catalyst (PTC). To produce disubstituted tetrazole [**(1c‐2c/1d‐2d)‐(1j‐2j)**], it is necessary to prepare several 5‐substituted tetrazoles (**1b‐2b**), which are prepared by combining various organic aldoximes with sodium azide in copper acetate Cu(OAc)_2_ in DMF as a solvent [26, 27]. Within 12 h of adding NaN_3_, aldoximes converted readily to the desired product in excellent yield. The 4‐(3‐chloropropyl)−3,6‐di(pyridin‐2‐yl)pyridazine was synthesized at reflux in toluene *N,N*‐Diméthylformamide using the reverse Diels‐Alder reaction between 3,6‐bis (2′‐pyridyl)−1,2,4, 5‐tetrazine and 5‐chloropentyne [34, 35, 36] (Scheme 2). After purifying on a silica gel column, two products were obtained (4‐(3‐chloropropyl)−3,6‐di(pyridin‐2‐yl)pyridazine and (E)‐N’‐(pyridin‐2‐ylmethylene)picolinohydrazide (**G**). A mauve crystal form of the 4‐(3‐chloropropyl)−3,6‐di(pyridin‐2‐yl)pyridazine compound was obtained with a 91% yield, while a white crystal form with a yield of 3% was obtained with the secondary compound (**G**). Although this low content does not preclude NMR analyses, the structure of this compound was determined only by X‐ray analysis. The 2‐chloro‐*N*,*N*‐diethylethylamine hydrochloride and 1‐(2‐chloroethyl)piperidine hydrochloride are commercially available. To alkylate various 5‐substituted tetrazoles and describe new disubstituted tetrazole synthesis [**(1c‐2c/1d‐2d)‐(1j‐2j)**], 4‐(3‐chloropropyl)−3,6‐di(pyridin‐2‐yl)pyridazine and 2‐chloro‐*N,N*‐diethylethylamine hydrochloride will be used to alkylate tetrazoles. These products were obtained by using DMF in conjunction with PTC for 12 h at room temperature [26, 27], as a step in Scheme 2. During the alkylation of tetrazoles 5‐substituted with 2‐chloro‐*N,N*‐diethylethylamine hydrochloride, we obtained two compounds when N‐alkylation took place at the N1 and N2 positions on the tetrazole ring, resulting in 1,5 (**1c‐2c**) minority tetrazoles and 2,5‐ (**1d‐2d**) disubstituted tetrazoles, another majority isomer, yielding 95% to 98% of each, respectively. Meanwhile, we obtained a single isomer 2.5 (**1j‐2j**) from the alkylating agent 4‐(3‐chloropropyl)−3,6‐di(pyridin‐2‐yl)pyridazine, which could be explained by the steric hindrance caused by the 3,6‐di(pyridin‐2‐yl)pyridazine group, which directs alkylation toward a single regioisomer when alkylated. The structures of these two regioisomers were determined using literature reports [26, 27, 37] and comparative ^1^H and ^13^C NMR spectroscopic analyses. The proposed structures of compounds **G** and **1j** were confirmed by X‐ray diffraction.

### 2.2 X‐Ray Diffraction Analysis

**FIGURE 1:**
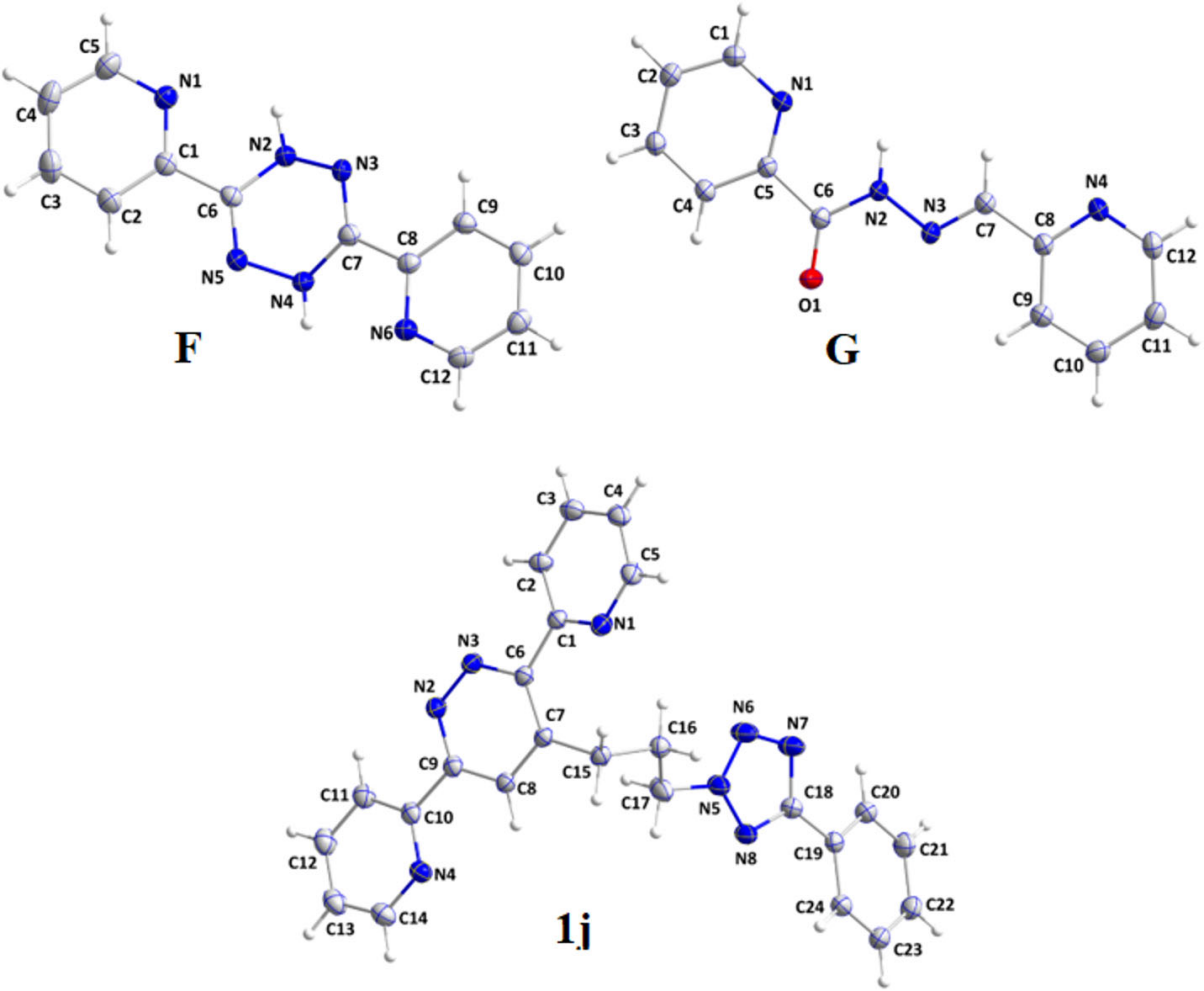
The asymmetric unit with labeling scheme and 50% probability ellipsoids for **F**, **G**, and **1j**.

This analysis was conducted to validate the **F**, **G**, and **1j** structures, as illustrated in Figure 1 and detailed in Table 1. Notably, the Orthorhombic system (*P*2_1_2_1_2_1_) crystallizes **F**, and the Monoclinic system crystallizes **G** and **1j** in P2_1_/n and *Cc* at the space group, respectively (Figure 1 and Table 1).

**TABLE 1:** Experimental details for **F**, **G**, and **1j**.

	F	G	1j
	Crystal data	Crystal data	Crystal data
Chemical formula	C_12_H_10_N_6_	C_12_H_10_N_4_O	C_24_H_20_N_8_
CCDC Deposition Number	2,531,191	2,531,190	2,531,188
F.Wt., g/mol	238.26	226.24	420.48
Crystal system	Orthorhombic	Monoclinic	
Space group	*P*2_1_2_1_2_1_	*P*2_1_/*n*	*Cc*
Temperature, K	150		
a, b, c, Å	7.2113 (1), 10.9932 (2), 13.8219 (3)	8.3516 (4), 13.9261 (6), 9.6410 (4)	21.9331 (8), 5.9211 (2), 15.8129 (6)
α, β, γ, °	—	— 104.769 (2) —	— 93.194 (1) —
V, Å^3^	1095.73 (3)	1084.25 (8)	2050.40 (13)
Z	4		
µ, mm^−1^	0.78	0.09	
Crystal size, mm	0.18 × 0.17 × 0.10	0.32 × 0.26 × 0.13	0.38 × 0.30 × 0.07
*Data collection*			
Diffractometer	Bruker D8 Venture PHOTON 3 CPAD		
No. of measured, independent, andobserved [I > 2σ(I)] reflections	29,880, 2,139, 2,126	50,371, 3,988, 3,516	56,921, 6,814, 6,452
R_int_	0.024	0.026	0.030
(sin θ/λ)max, Å^−1^	0.618	0.760	0.736
Refinement			
R[F^2^ > 2σ(F^2^)], wR(F^2^), S	0.022, 0.057, 1.06	0.045, 0.128, 1.08	0.037, 0.104, 1.05
No. of reflections	2,139	3,988	6,814
No. of parameters	171	158	289
Δρmax, Δρmin, e Å^−3^	0.13, −0.13	0.48, −0.23	0.33, −0.18

### 2.3 DFT Outputs

The investigation into the chemical reactivity of the synthesized compounds was conducted by analyzing known global and local indicators of reactivity. For this purpose, the DFT method was used to estimate these indicators in the gas phase. Table 2 presents the main results for selected energetic parameters related to the global reactivity of the developed compounds. It is recognized that the energy of the HOMO and LUMO‐type molecular orbitals has a key role in estimating the chemical reactivity of a given organic compound, as approved by K. Fukui [38]. According to the tabulated values, the HOMO energy levels are ordered as **F** < **G** < **1j**. This finding highlights the F molecule's ability to donate electrons during a potential chemical reaction, compared with **G** and **1j**. In this regard, the values of LUMO energy demonstrate the good ability of the same compound (i.e., **F**) to accept electrons efficiently during a chemical process. In contrast, the **G** compound is a poor acceptor of electrons. A smaller Δ*E* indicates greater chemical reactivity, as less energy is required to promote electrons from the HOMO to the LUMO during chemical reactions [39, 40]. In our study, the higher reactivity of the **F** molecule compared with other molecules is further confirmed by the calculated Δ*E* values, which are the lowest for the same compound (Δ*E* = 3.390 eV). Furthermore, this value is comparable to other biologically active tetrazole derivatives reported in the literature, where Δ*E* values typically range from 3.2 to 5.5 eV [41]. On the other hand, according to the hard and soft acids and bases (HSAB) theory, the softest molecule interacts well with soft materials, such as metal substrates [42]. As a quantum‐chemical indicator, the chemical hardness (*η* parameter) is primarily used to assess this intrinsic molecular property. The tabulated values of this indicator show that the hardness of investigated molecules follows the **F** < **1j** < **G** order, which means that **F** is the softer molecule than **1j** and **G.** The elevated global electrophilicity (*ω*) value of compound **F** confirms that this compound can form strong binding affinities with biological macromolecules through various interactions [43].

**TABLE 2:** Main calculated molecular electronic structure parameters for the newly developed compounds.

Molecule	*E*_HOMO,_ eV	*E*_LUMO,_ eV	Δ*E*, eV	*η*, eV	*χ*, eV	*μ*, eV	*ω*, eV	*ε*, eV^−1^
**F**	−6.167	−2.777	3.390	1.695	4.472	4.472	5.899	0.170
**G**	−6.283	−2.026	4.257	2.128	4.154	4.154	4.054	0.247
**1j**	−6.374	−2.141	4.233	2.116	4.257	4.257	4.281	0.234

**FIGURE 2:**
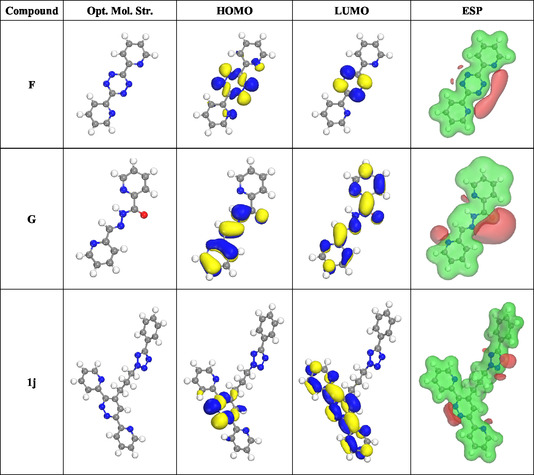
Relaxed molecular structures of **G**, **F,** and **1j** compounds with their associated molecular frontier orbitals (HOMO and LUMO) and ESP maps (red and green colors refer to electronegative and ‐positive potentials).

Regarding the local reactivity of the investigated molecules, we examine it by analyzing the distribution of frontier orbital density. ESP maps over molecules as depicted in Figure 2. As can be noted, the repartition of the more probable regions for the electron‐donating process, that is, corresponding to the HOMO‐type orbital, is mainly located in the middle of the G and 1j molecule backbone. At the same time, it was more focused on the skeleton of the **F** molecule. Concerning the LUMO‐type orbitals, it can be observed that the electron‐gain process in a given system is likely to arise via the almost molecular backbone of the F molecule, followed by the 1j and then the G orbitals. On the other hand, observations of ESP maps for the three studied compounds show that the presence of additional heteroatoms affects their distribution. The regions with negative potentials are located around the heteroatom. At the same time, positive potentials characterize the rest of the molecular backbone. Furthermore, the positive and negative potential regions refer to the electron‐poor and electron‐rich regions within the molecule, respectively. For instance, the investigated molecules can interact electrostatically with a given positively charged material (or molecules) via these electron‐rich regions [44, 45, 46, 47].

### 2.4 Monte Carlo/SAA Simulations

**FIGURE 3:**
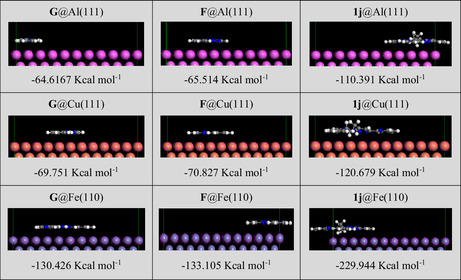
Side view of the most equilibrium configurations of **G**, **F,** and **1j** compounds on different considered metal surfaces with their associated adsorption energies.

To anticipate the potential inhibitory effect of the synthesized compounds on metal corrosion, Monte Carlo/SAA simulations were performed on aluminum, copper, and iron. Figure 3 shows the relaxed adsorption geometries for different compound@metal systems, along with their corresponding adsorption energies. As noted, a parallel adsorption configuration was observed for the three investigated molecules on all metal surfaces. In this regard, it is recognized that a flat adsorption geometry of a given inhibitor molecule can provide maximum coverage of the target metal surface, thereby limiting the free surface for potential attack by corrosive species present in the solution. In our case, this can be explained by the presence of numerous favorable adsorption sites along the molecular backbone of the studied molecules, including nitrogen and oxygen heteroatoms and double bonds [48]. Based on the value of the adsorption energies, one can conclude that the three developed compounds show apparent tendencies (i.e., spontaneous process) to be adsorbed on all studied metal surfaces. Furthermore, it can be shown that the molecular structure of these compounds and the nature of the target metal surface were also meaningfully influenced by the magnitude of the adsorption energy, thereby affecting the adhesion of the protective layer to the metal surface. Without exception, the absolute values of adsorption energies are ordered as follows: Fe(110) > Cu(111) > Al(111) for all examined molecules. This outlines the improved ability of these molecules to adsorb onto iron rather than copper or aluminum. Therefore, it can be expected that a reasonable protection capacity for these compounds can be achieved in the first stage for iron, followed by copper and aluminum metals [49]. On the other hand, the compound **1j** exhibits the more negative adsorption energy, that is, a higher tendency to adsorb overall selected metal surfaces in comparison to **F** and **G** molecules. Hence, actual Monte Carlo/SAA simulations expected compound **1j** to be the more effective inhibitor vis‐à‐vis other studied compounds.

### 2.5 Molecular Docking Studies

The conformation with the lowest Vina score was considered the most stable and was further analyzed for its interactions. The hydrogen and hydrophobic interaction details of all docking‐obtained conformations from Vina Ligand Gen are analyzed, and the ligands that showed a better Vina score, along with their interactions, are further considered for dynamics simulation [50, 51, 52]. It is observed that the reference ligand PD180970 (PDB 2HZI) forms one hydrogen bond with Met318, with a Vina score of −9. The co‐crystallized inhibitor AXI (PDB 4TWP) forms two hydrogen bonds with Tyr253 and Met318, with a Vina score of −8.9. All interactions match those available in the PDBSum, validating the docking tool and using the Vina score as a reference. Ligand 3D exhibited a Vina score of −8.5 and formed a hydrogen bond with Gly249 of the 2HZI protein. Ligand 2J exhibited the lowest Vina score (−8.3) and formed a hydrogen bond with Tyr253 of 4TWP. All ligand complexes were subsequently selected for dynamics simulation (Table 3).

**TABLE 3:** Vina score, hydrogen bonds, hydrophobic interactions, LigPlots, and 3D interaction for the ligands considered for dynamics simulation.

Protein	Ligand	Vina score	Hydrogen bonds	2D interaction	3D interaction
2HZI	**3d**	−8.5	Gly321	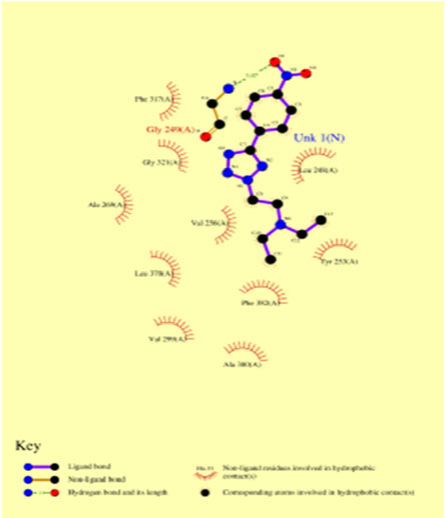	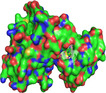
	**JIN**	−11.7	Met318	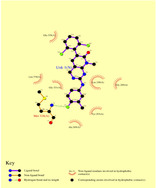	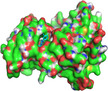
4TWP	**2j**	−8.3	Tyr253	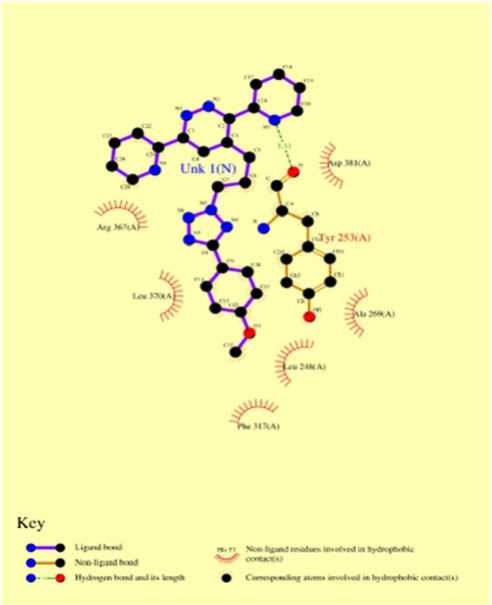	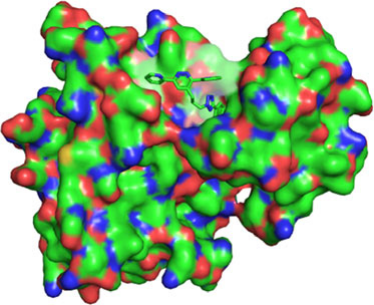
	**Axitinib**	−8.9	Tyr253 | Met318	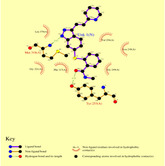	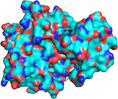

### 2.6 Molecular Dynamics Simulation

The RMSD plot indicates that ligand JIN reduces overall deviation from the unbound 2HZI protein. Reduced RMSF fluctuation in positions 70–80, 160−170, and 210−225 suggests that ligand JIN may stabilize this region. Strong hydrogen interaction at MET318, hydrophobic interaction at MET318, and TYR253 enhance complex stability. The RMSD plot indicates that ligand **3d** maintains stability similar to the unbound 2HZI protein, except between 20 and 40 ns, where it reduces the deviation. Reduced RMSF fluctuation in positions 70–80, 160−170, and 210−225 suggests ligand **3d** may stabilize this region, with a strong Hydrogen interaction at TYR253 and ASP381. A Hydrophobic interaction at TYR253 was observed, which enhances complex stability. Overall, ligand 3d exhibits stability to the unbound protein, with localized stabilization in positions 70–80, 160−170, 210−225, and strong protein–ligand interactions Table 4.

**TABLE 4:** RMSD, RMSF, Interaction fraction and ligand–protein contacts between JIN and 3d with 2HZI.

2HZI	
**RMSD and RMSF**	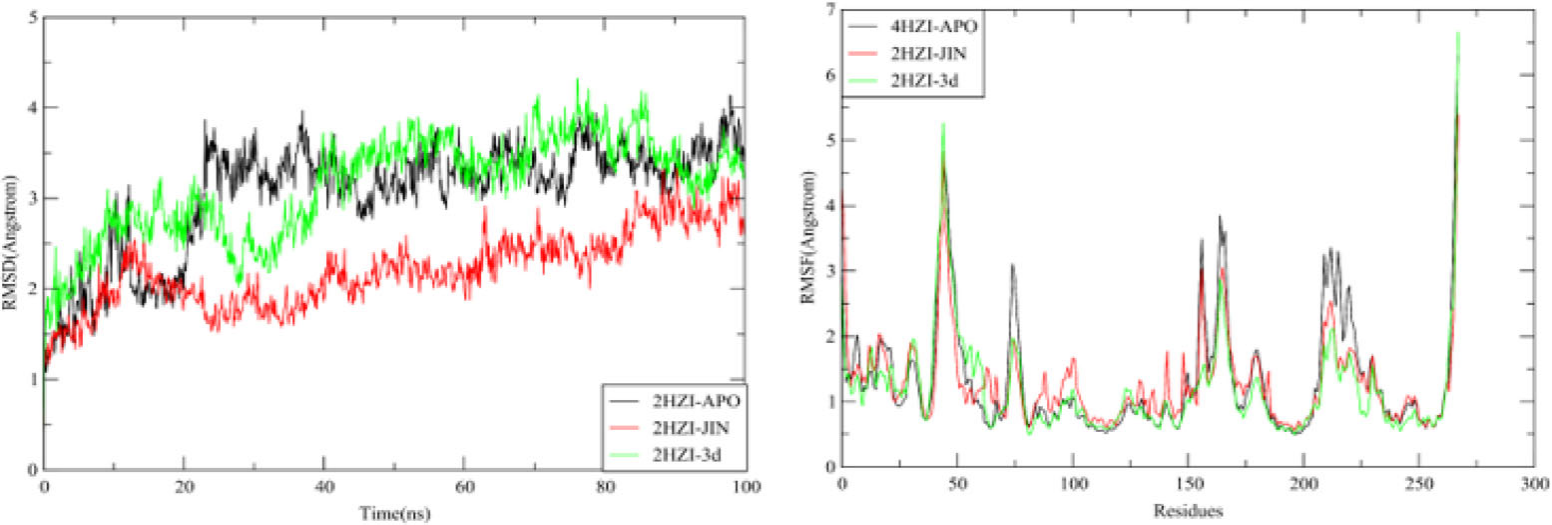
**Interaction (JIN)**	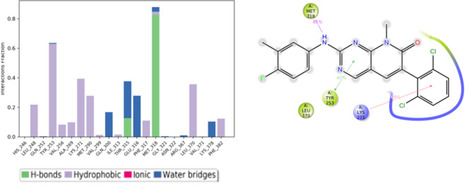
**Interaction (3d)**	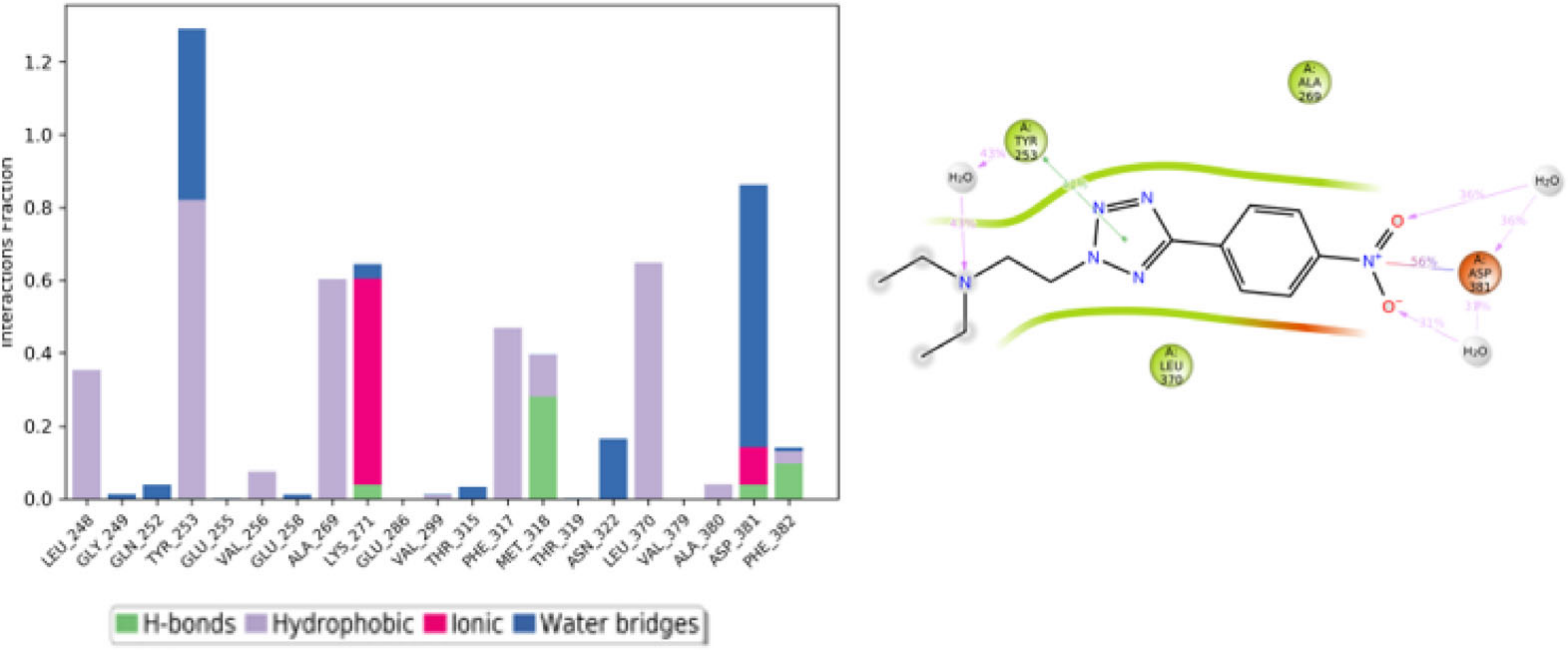

The RMSD plot indicates that ligand AXI maintains stability similar to that of the unbound 4TWP protein. Reduced RMSF fluctuation in positions 15–25 and 200−220 suggests that ligand AXI may stabilize this region. Strong hydrogen bonding at GLU316, hydrophobic interactions at MET318 and TYR253, and a water bridge at TYR253 all contribute to enhancing complex stability. The RMSD plot indicates that ligand **2j** initially exhibits a significant deviation; however, after 80 ns, it maintains stability similar to that of the unbound 4TWP protein. Reduced RMSF fluctuation in positions 15–25 and 200−220 suggests that ligand **2j** may stabilize this region. Strong hydrogen‐bonding interactions were observed at MET318, and hydrophobic interactions were observed at TYR253, PHE317, MET318, and LEU370, thereby enhancing complex stability. Overall, ligand **2j** exhibits stability to the unbound protein, with localized stabilization in positions 15–25, 200−220, and strong protein–ligand interactions Table 5.

**TABLE 5:** RMSD, RMSF, Interaction fraction, and ligand–protein Contacts between AXI and 2j with 4TWP.

4HZI	
**RMSD and RMSF**	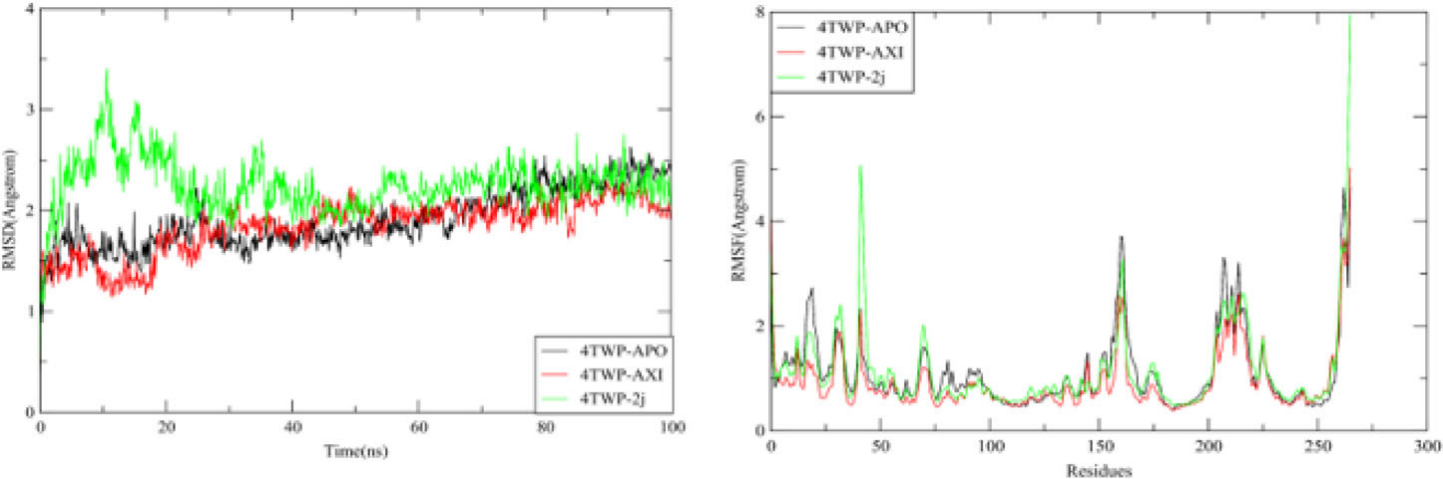
**Interaction (AXI)**	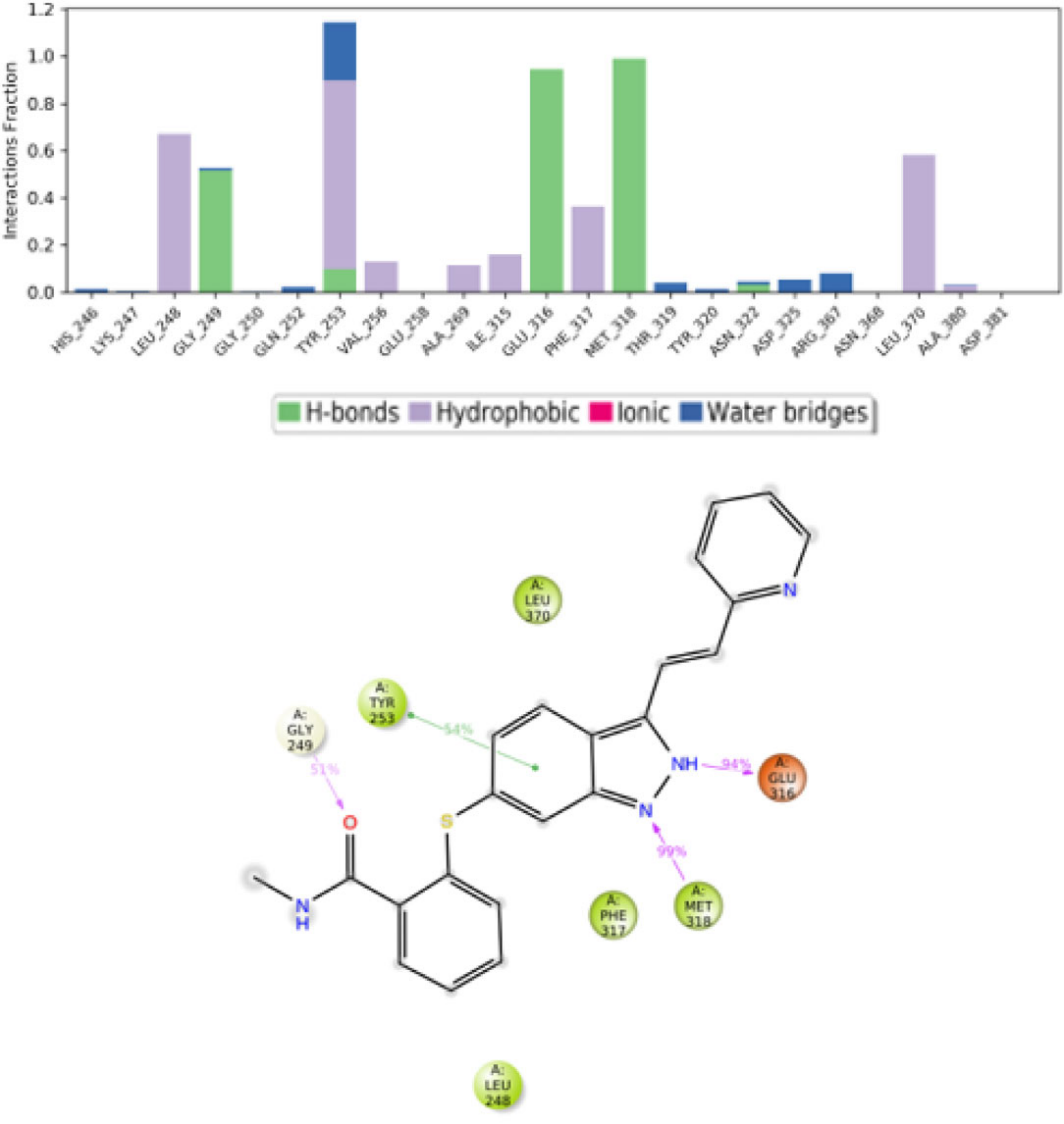
**Interaction (2j)**	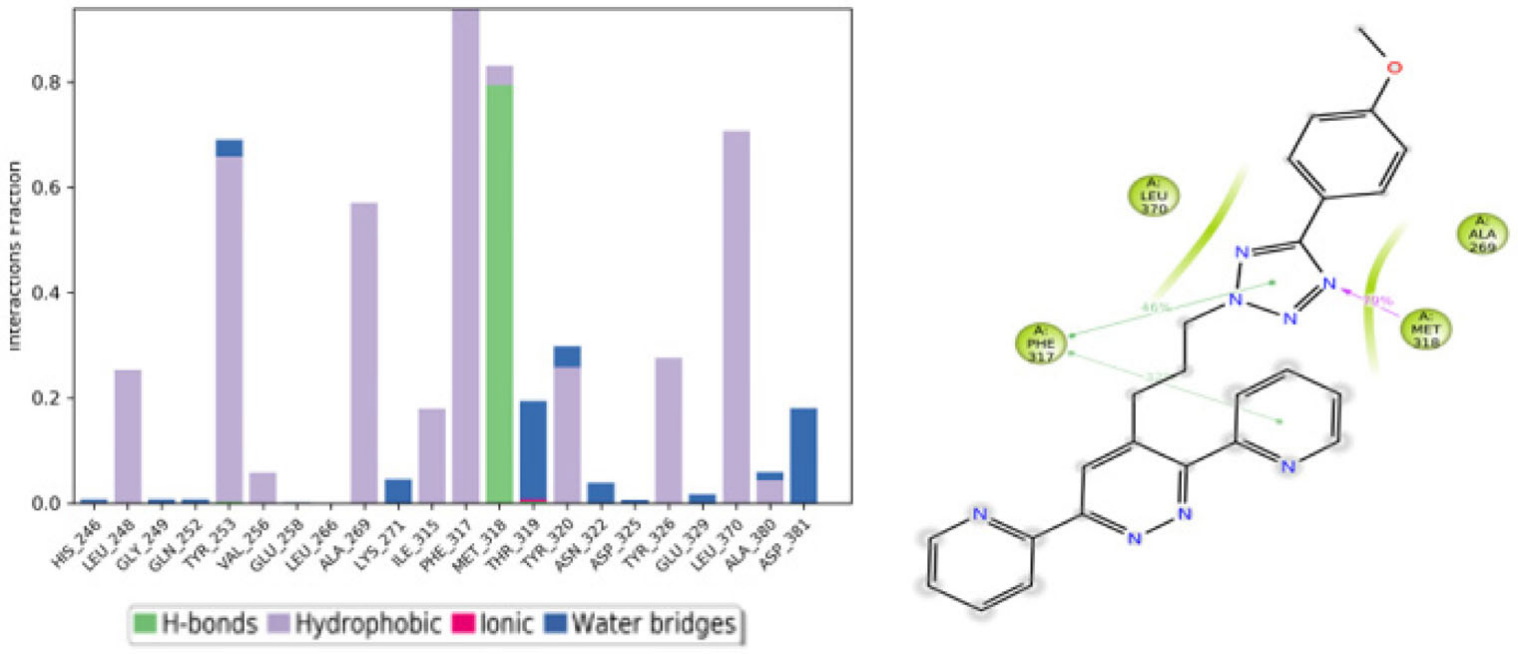

## 3 Conclusion

In this work, new 1,5‐ and 2,5‐disubstituted tetrazole derivatives were successfully synthesized under phase‐transfer catalysis conditions with moderate to excellent yields and characterized using spectroscopic techniques and single‐crystal X‐ray diffraction analysis. The regioselectivity of the alkylation reactions was found to be strongly influenced by steric and electronic effects of the substituents. DFT calculations provided valuable insight into the structural and electronic properties of the synthesized compounds, where compound **F** exhibited the highest chemical reactivity due to its lower HOMO‐LUMO energy gap. Electrostatic potential analyses further revealed the preferential reactive sites involved in intermolecular interactions. Monte Carlo/SAA simulations demonstrated strong adsorption affinities of the synthesized compounds toward Al(111), Cu(111), and Fe(110) metallic surfaces, particularly for compound **1j**, suggesting promising corrosion inhibition behavior. Molecular docking investigations against Abl kinase targets (2HZI and 4TWP) revealed favorable binding affinities and significant interactions with key active‐site residues. Furthermore, 100 ns MD simulations confirmed the stability of the selected protein–ligand complexes throughout the simulation time. Overall, the integration of experimental synthesis, structural characterization, theoretical calculations, corrosion inhibition prediction, and biological evaluation highlights the multifunctional potential of these tetrazole derivatives and provides a useful platform for the future development of biologically active and industrially relevant heterocyclic compounds.

## 4 Materials and Methods & Experimental Section

### 4.1 Materials and Methods

We used an IA 9000 electrothermal unit or a Köfler heating bench to determine the melting points of all the synthesized compounds. NMR spectra were recorded in deuterated CDCl3 solutions on a Bruker Avance DPX 300 spectrometer. During the analysis, tetramethylsilane, with a chemical shift of 0 ppm, was used as a reference, and chemical shifts were reported in parts per million (ppm). J is expressed in Hertz (Hz), and s (singlet), d (doublet), dd (doublet doublet), t (triplet), t (triplet doublet), q (quadruplet), and m (multiple) correspond to signal multiplicity. Except for any special instructions, almost all commercially available solvents and chemicals were readily available. Silica gel plates (Merck 60, F254) and silica gel (Merck 60, 230–400 mesh) were used for both thin‐layer and column chromatography (TLC).

### 4.2 General Procedure for Synthesis of Disubstituted Alkyl Tetrazoles ((1c‐4c/1d‐4d)‐(1j‐2j))

#### 4.2.1 Structural Characterization of *N,N*‐Diethyl‐2‐(5‐(4‐Methoxyphenyl)‐1H‐Tetrazol‐1‐Yl)Ethanamine (2c)

**FIGURE 4:**
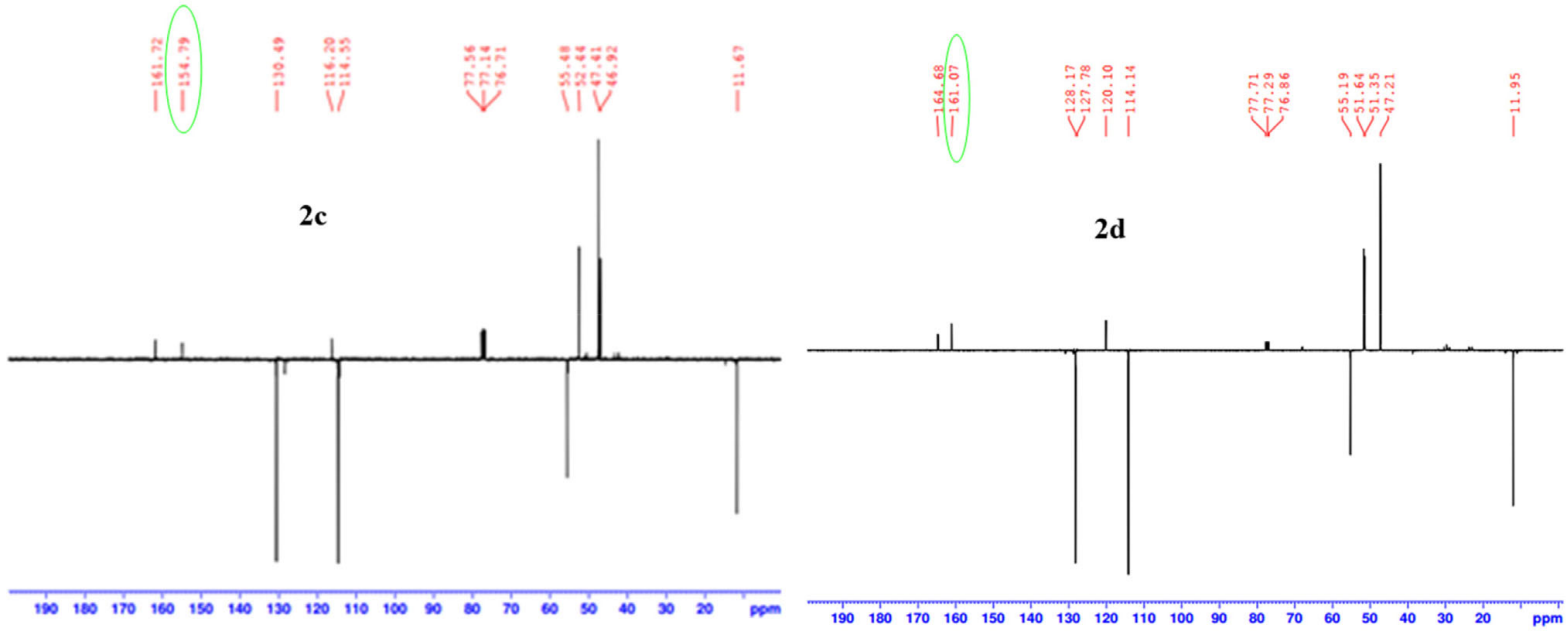
These ^13^C NMR spectra are for compounds **2c** and **2d**, respectively.

Based on the ^1^H NMR spectrum (Figure 4) of compound **2c**, the six aliphatic CH_3_ protons on the two carbon chains produce a triplet at 0.83 ppm, a singlet at 3.86 ppm due to the methoxy group, and an A_2_B_2_ system resulting from two doublets corresponding to aromatic nucleus protons around 7.02 and 7.69 ppm. According to the ^13^C NMR spectrum of compound **2c** (Figure 4), a signal at 11.67 ppm indicates that there are two carbons in the methyl groups of two ethyl chains, a signal at 52.48 ppm indicates that a methoxy group is present, and a signal at 154.79 ppm indicates that isomer 1.5 is present. Despite the similarities between compound 4d and compound 4c, the ^1^H NMR spectrum of compound **2d** has a change in chemical shift, with the protons of the methylene groups resonating at 2.99 and 4.58 ppm in regioisomer 2, 5 instead of 2.92 and 4.41 ppm in regioisomer 1,5. A ^13^C NMR spectrum of compound **2d** (Figure 4) shows a powerful signal at 164.68 ppm associated with the carbon of the tetrazole ring (isomer 2,5). It was indeed possible to distinguish between 1,5‐ and 2,5‐disubstituted regioisomers by the characteristic chemical shift of the C5 atom in tetrazole. In the 2,5‐tetrazole compound disubstituted (**2d**), the C5 atom is displaced by about 10 ppm compared to the 1,5‐disubstituted 4 regioisomer.

#### 4.2.2 Structural Characterization of Compound 1j

Upon analyzing the ^1^H NMR spectrum of product **1j**, we found nine signals between 7.30 and 8.74 ppm attributable to the aromatic protons. In addition, the signals showing the six protons of the three methylene functions linking the nucleus of the tetrazole to the 3,6‐di(pyridin‐2‐yl)pyridazine were found to be between 2.51, 3.24 and 4.76 ppm, thus confirming N‐alkylation. Using ^13^C NMR, we detect 19 signals between 121.77 and 149.45 ppm due to aromatic carbons, as well as a signal at 165.11 ppm due to a quaternary carbon, thus confirming the formation of a single isomer of tetrazole 2,5‐disubstituted.

### 4.3 Spectral Data

#### 4.3.1 Synthesis of 4‐(3‐Chloropropyl)‐3,6‐Di(Pyridin‐2‐Yl)Pyridazine and (E)‐N’‐(Pyridin‐2‐Ylmethylene)Picolinohydrazide

In a flask, dissolve **4** mmol of 3,6‐bis(2‐pyridyl)−1,2,4,5‐tetrazine in 20 mL of DMF. Then, add 1 equivalent of 5‐chloropentyne and leave the reaction mixture to reflux in the DMF under stirring. TLC monitored the reactions. Once the responses were complete, the solvent was evaporated. The products are obtained in pure form after silica gel column chromatography using a hexane/ethyl acetate eluent (1:5).

##### 4‐(3‐Chloropropyl)−3,6‐Di(pyridin‐2‐Yl)pyridazine: H

**Yield (%)** = 91; m*p* = 85 °C; Rf: 0.3 (hexane/ethyl acetate: 1:3 v/v), ^**1**^ **H NMR (300 MHz, CDCl3)**: 2,13–2,22 (m, 2H, CH2); 3,23–3,22 (td, 2H, CH2); 3,53–3,29 (td, 2H, CH2); 7,4 (m, 2H, CH_ar_); 7,9 (td, 2H, CH_ar_); 8,2 (d, 1H, CH_ar_); 8,5 (s, 1H, CH_ar_); 8,70–8,75 (m, 3H, CHar). ^**13**^ **C NMR (75 MHz, CDCl3)**: 30,16(CH2‐Cl); 32,39; 44,49 (2CH2); 121,75; 123,72; 124,72; 124,76; 125,94; 136,95; 137,19; 148,58; 149,43 (CH_ar_); 141,51; 153,36; 156,05; 157,32; 158,83 (Car) (see Figures S1, S2 and S3).

#### 4.3.2 Syntheses of [(1c‐2c/1d‐2d)‐(1j‐2j)] via N‐Alkylation Reaction Under PTC Conditions

In 20 ml of DMF, dissolve (1 mmol) 5‐substituted tetrazole and (1 mmol) (2‐chloro‐*N,* *N*‐diethylethylamine hydrochloride and 4‐(3,4‐chloropropyl)−3,6‐di(pyridin‐2‐yl)pyridazine) and then add (2 mmol) potassium carbonate and (0.20 mmol) tetra‐n‐butyl ammonium bromide. to the mixture. Following 12 h of reflux in DMF, the reaction mixture was evaporated using a rotary evaporator with distilled water, before dichloromethane was added to extract the residual product. A reduced‐pressure evaporator is used to reduce the pressure of the organic phase after it is dried with Na_2_SO_4_. Following purification of the residue using silica column chromatography, it was eluted with a hexane/ethyl acetate mixture (9/1) and then recrystallized in ethanol.

#### *N,N*‐Diethyl‐2‐(5‐(4‐Nitrophenyl)‐1H‐Tetrazol‐1‐Yl)ethanamine: 1c

**Yield (%)** = 21%; Rf: 0.53 (hexane/ethyl acetate: 4:1 v/v), ^**1**^ **H NMR (300 MHz, CDCl**_**3**_ **)**: 0.97 (m, 6H, 2 CH_3‐aliphatic_); 2.54 (q, 4H, 2CH_2_, *J* = 7.2); 3.06 (t, 2H, CH_2_, *J* = 6.6/13.5); 4.66 (t, 2H,CH_2_, *J* = 6.9/13.8); 8.35(s, 4H, 4CH_‐Ar_).^**13**^ **C NMR (75 MHz, CDCl**_**3**_ **)**: 11.40(2CH_3‐aliphatic_); 47.30, 47.96, 51.56, 51.76 (4 CH_2_‐N); 125.68 (2CH_‐Ar_); 126.34 (2CH_‐Ar_); 128.82(Cq_‐Ar_); 129.45(Cq_‐Ar_); 154.70 (Cq‐_1.5T_) (see Figures S4 and S5).

#### *N,N*‐Diethyl‐2‐(5‐(4‐Nitrophenyl)‐2H‐Tetrazol‐2‐Yl)ethanamine: 1d

**Yield (%)** = 75%; **mp** = 97 °C; Rf : 0.61 (hexane/ethyl acetate: 4:1 v/v), ^**1**^ **H NMR (300 MHz, CDCl**_**3**_ **)**: 1.01 (t, 6H, 2 CH_3‐aliphatic_, *J* = 6.9); 2.60 (q, 4H, 2CH_2_, *J* = 7.2); 3.13 (t, 2H, CH_2_, *J* = 6.9); 4.75 (t, 2H,CH_2_, *J* = 6.9); 8.35(s, 4H, 4CH_‐Ar_). ^**13**^ **C NMR (75 MHz, CDCl**_**3**_ **)**: 12.02(2CH_3‐aliphatic_); 47.32, 51.77, 51.96 (4 CH_2_‐N); 124.21(2CH_‐Ar_); 127.62 (2CH_‐Ar_); 133.48(Cq_‐Ar_); 148.80(Cq_‐Ar_); 163.07 (Cq‐_2.5T_) (see Figures S6 and S7).

#### *N,N*‐Diethyl‐2‐(5‐(4‐Methoxyphenyl)‐1H‐Tetrazol‐1‐Yl)ethanamine: 2c

**Yield (%)** = 36%; Rf: 0.58 (hexane/ethyl acetate: 4:1 v/v), ^**1**^ **H NMR (300 MHz, CDCl**_**3**_ **)**: 0.83 (t, 6H, 2 CH_3‐aliphatic,_ *J* = 6.9, 7.2); 2.42 (q, 4H, 2CH_2_, *J* = 7.2); 2.92 (t, 2H, CH_2_, *J* = 6.3) 3.86 (s, 3H, OCH_3_); 4.41 (t, 2H, CH2, *J* = 6.6); 7.02(d, 2H, 2CH_‐Ar_, *J* = 9); 7.69(d, 2H, 2CH_‐Ar_, *J* = 9). ^**13**^ **C NMR (75 MHz, CDCl**_**3**_ **)**: 11.67 (2CH_3‐aliphatic_); 46.92, 47.41, 52.44(4 CH_2_‐N); 44.48(OCH_3_); 114.55 (CH_‐Ar_); 116.20(Cq_‐Ar_); 130.49 (CH_‐Ar_); 154.79 (Cq‐_1.5T_); 161.72 (Cq_‐OCH3_) (see Figures S8 and S9).

#### *N,N*‐Diethyl‐2‐(5‐(4‐Methoxyphenyl)‐2H‐Tetrazol‐2‐Yl)ethanamine: 2d

**Yield (%)** = 61%; **mp** = 98 °C; Rf: 0.64 (hexane/ethyl acetate: 4:1 v/v), ^**1**^ **H NMR (300 MHz, CDCl**_**3**_ **)**: 0.83 (t, 6H, 2 CH_3‐aliphatic_, *J* = 7.2); 2.42 (q, 4H, 2CH_2_, *J* = 7.2); 2.92 (t, 2H, CH_2_, *J* = 6.3) 3.86 (s, 3H, OCH_3_); 4.41 (t, 2H, CH2, *J* = 6.9); 6.89 (d, 2H, 2CH_‐Ar_, *J* = 8.7); 7.99 (d, 2H, 2CH_‐Ar_, *J* = 8.7). ^**13**^ **C NMR (75 MHz, CDCl**_**3**_ **)**: 11.95 (2CH_3‐aliphatic_); 47.21, 51.35, 51.64 (4 CH_2_‐N); 55.19 (OCH_3_); 114.14 (CH_‐Ar_); 120.10 (Cq_‐Ar_); 128.17 (CH_‐Ar_); 161.07 (Cq_‐OCH3_); 164.68 (Cq‐_2.5T_) (see Figures S10 and S11).

#### 4‐(3‐(5‐Phenyl‐2H‐Tetrazol‐2‐Yl)propyl)−3,6‐Di(pyridin‐2‐Yl)pyridazine: 1j

**Yield (%)** = 97%; **mp** = 117 °C; Rf: 0.53 (hexane/ethyl acetate: 4:1 v/v), ^**1**^ **H NMR (300 MHz, CDCl**_**3**_ **)**: 2.51 (qt, 2H, CH_2_); 3.24 (t, 2H, CH_2_, *J* = 8.1); 4.76 (t, 2H, CH_2_, *J* = 13.5); 7.30 (dd, 1H, CH_ar_, *J* = 5.4,6); 7.41 (dd, 1H, CH_ar_, *J* = 5.1, 7.2); 7.50 (m, 3H, CH_ar_); 7.89 (m, 2H, CH_ar_); 8.15 (dd, 2H, CH_ar_, *J* = 2.1, 5.4); 8.23 (d, 1H, CH_ar_, *J* = 7.8); 8.544 (s, 1H, CH, CH_ar_); 8.73 (dd, 1H, CH_ar_, *J* = 0.9, 1.8); 8.74 (dd, 2H, CH_ar_, *J* = 0.9,3.9). ^**13**^ **C NMR (75 MHz, CDCl**_**3**_ **)**: 29.80, 29.92, 52.77(3 CH_2_); 121.77, 123.74, 124.73, 124.77, 126.06, 126.84, 127.44, 128.88, 130.28, 136.95, 137.19, 148.54, 149.45 (CH_ar_); 127.44 (Cq); 140.52 (Cq); 153.30 (Cq); 155.88(Cq); 157.38(Cq); 158.54(Cq); 165.11(Cq‐_tétrazole_) (see Figures S12 and S13).

#### 4‐(3‐(5‐Phenyl‐2H‐Tetrazol‐2‐Yl)propyl)−3,6‐Di(pyridin‐2‐Yl)pyridazine: 2j

**Yield (%)** = 94%; **mp** = 125 °C; Rf: 0.52 (hexane/ethyl acetate: 4:1 v/v), ^**1**^ **H NMR (300 MHz, CDCl**_**3**_ **)**: 2.32 (m, 2H, CH_2_); 3.24 (t, 2H, CH_2_, *J* = 6.9); 3.85 (s, 3H, OCH_3_); 4.72 (t, 2H, CH_2_, *J* = 9/13.2); 6.95 (dd, 1H, CH_ar_, *J* = 8.7); 7.33 (dd, 1H, CH_ar_, *J* = 12); 7.42 (m, 2H, CH_ar_); 7.87 (m, 2H, CH_ar_); 7.91 (m, 3H, CH_ar_, *J* = 2.1, 5.4); 8.06 (d, 2H, CH_ar_, *J* = 8.7); 8.18 (dd, 1H, CH, Ch_ar_, *J* = 8.7); 8.55 (m, 1H, CH_ar_), 8.73 (m, 2H, CH_ar_). ^**13**^ **C NMR (75 MHz, CDCl**_**3**_ **)**: 28.92, 29.18 (2 CH_2_); 52.67(CH_2_); 53.47(OCH_3_); 120.00, 122.58, 123.99, 124.47, 125.88, 128.80, 130.91, 132.43, 140.59, 141.73, 149.41 (Cq); 155.82 (Cq); 156.16 (Cq); 158.56(Cq); 158.94(Cq); 161.22 (Cq); 163.34(Cq); 164.98(Cq); 166.20(Cq_−2.5T_) (see Figures S14 and S15).

### 4.4 X‐Ray and Theoretical Studies

The X‐ray intensity data for compounds **F**, **G,** and **1j** were collected using a Bruker D8 VENTURE PHOTON 3 CPAD diffractometer equipped with an INCOATEC IμS‐Cu microfocus source. Data frames were integrated with the Bruker SAINT software package [53] using a narrow‐frame algorithm, while empirical absorption corrections and merging of equivalent reflections were performed with SADABS [54]. The crystal structures were solved using SHELXT, and all non‐hydrogen atoms were refined anisotropically on F^2^ by full‐matrix least‐squares methods with SHELXL [55, 56]. The crystallographic and experimental details for compounds **F**, **G,** and **1j** are summarized in Table 1.

### 4.5 Computational Studies Details

#### 4.5.1 1 DFT Calculations

To explore the global and local reactivity of developed compounds, DFT calculations were performed using the B3LYP hybrid functional with the DNP 3.5 basis set in the gas phase. The global reactivity was limited in the computation of higher occupied molecular orbital and lower unoccupied molecular orbital energies (E_HOMO_ and E_LUMO_, respectively), as well as their corresponding gap energy (Δ*E*_gap_), electronegativity (*χ*), chemical hardness (η), chemical potential (μ), electrophilicity (ω), and nucleophilicity index (*ε*). Whereas the local one was performed by analyzing the distribution of frontier molecular orbitals and electrostatic potential (ESP) maps [57, 58, 59]. During the geometry relaxations, the convergence tolerances are fixed at 1 × 10^–5^ Ha, 2 × 10^–3^ Ha Å^−1,^ and 5 × 10^–3^ Å for energy change, gradient, and displacement, respectively.

#### 4.5.2 Monte Carlo/SAA Simulations

To account for the potential inhibitory effect of the developed compounds against metal corrosion, Monte Carlo simulations coupled with the simulated annealing algorithm (SAA) are performed [60, 61]. The aluminum (Al), copper (Cu), and iron (Fe) substrates are selected as target metals for this predictive study. For this, the simulation boxes with the following 3D dimensions, including 60 Å as a vacuum region, are adopted: 28.6 × 28.6 × 69.3 Å, 28.1 × 28.1 × 68.3 Å and 27.3 × 27.3 × 68.1 Å for Al(111), Cu(111), and Fe(110) substrates. Five cycles of heating/cooling with 5 × 10^4^ steps are used to conduct these simulations, using COMPASS‐II as the force field to calculate all energy terms. The convergence tolerances are fixed at 1 × 10^–4^ Ha, 5 × 10^–3^ Ha Å^−1^ and 5 × 10^–5^ Å for energy, force, and displacement. The Ewald and atom‐based summation methods are applied to estimate the electrostatic and Van der Waals interactions [62].

### 4.6 Molecular Docking Studies

#### 4.6.1 Protein Preparation

The 3‐dimensional crystal structures of proteins were retrieved from the RCSB PDB (Research Collaboratory for Structural Bioinformatics PDB). The Abl kinase domain in complex with PD180970 (PDB ID: 2HZI) and the crystal structure of the human ABL1 T315I gatekeeper mutant kinase domain in complex with axitinib (PDB ID: 4TWP) were downloaded in .pdb format. The bound ligands 6‐(2,6‐dichlorophenyl)‐2‐[(4‐fluoro‐3‐methylphenyl)amino]‐8‐methylpyrido[2,3‐d]pyrimidin‐7(8h)‐one (JIN) in 2HZI and Axitinib (AXI) in 4TZK were removed. The proteins were converted to PDBQT using PyRX 0.8.

#### 4.6.2 Ligand Preparation

To validate the docking tool and to obtain the interaction score, the co‐crystallized ligands were redocked. The successful scoring function, as defined by the validation technique described in the literature, is the one where the RMSD of the best docked conformation is less than 2 Å from the experimental one. Each synthetic molecule was drawn in ACD Chem Sketch and saved as a .mol file. The Open Babel program was used to convert all the molecules drawn into PDB format, and the PRODRG server was used for energy minimization.

#### 4.6.3 Molecular Docking

For docking studies, the AutoDock Vina program (Trott et al. (2010)) [63] was used. It is an efficient tool for predicting protein–ligand binding. AutoGrid, as described by Morris et al. (2008) [64], was used to define protein docking areas. The grid box size of 52 × 54 × 65 Å and centered at x, y, and z coordinates of 19.25, 12.28, 14.83 for 2HZI, grid box size of 79 × 59 × 32 and centered at x, y, and z coordinates of 69.46, 11.63, 46.16 for 4TWP, respectively, for all the ligands, including reference to the target proteins. The AutoDock Vina exhaustiveness of 8 was applied using PyRx 0.8, a Graphical User Interface for docking studies. Vina LigGen was used to generate a Ligand Plot for each conformation [65, 66].

### 4.7 Molecular Dynamics Simulation

MD simulations were performed on both proteins, considering their APO form and the protein‐ligand complexes identified after docking using Desmond Maestro v11.3 [67]. Initially, the Protein Preparation Wizard was used to pre‐process the protein, and the System Builder utility was used to prepare the system for simulation. The protein was then placed individually in an orthorhombic simulation box, with a distance of 10 Å from the edges. The system was solvated using a pre‐equilibrated simple point charge (SPC) water model, and a 0.15 M salt (NaCl) concentration was added, along with Na^+^ and Cl^−^ ions, to neutralize the system. Before MD simulations, the model system was relaxed using the standard Desmond protocol. This is a six‐step relaxation protocol, which includes an initial short simulation of 12 ps in NVT ensemble at *T* = 10 K with restraints on solute heavy atoms, a 12 ps simulation in NPT ensemble with *T* = 10K and pressure (*P*) = 1 atm restraining the solute heavy atoms, a 12 ps simulation in NPT ensemble with restraints on nonhydrogen solute atoms, and a 24 ps simulation in NPT ensemble without restraints. The pressure and temperature conditions were stabilized using the isobaric and isothermal ensemble with the Martyna‐Tobias‐Klein barostat and Nosé–Hoover thermostat, which are the default protocols in Desmond. Finally, the OPLS3 [68] force field was used to assign force field parameters for the protein. A 100 ns MD simulation in the NPT ensemble (*T* = 300K and *p* = 1 atm) was performed, and the trajectory was recorded at 100 ps intervals, yielding approximately 1000 frames.

## Funding

The authors extend their appreciation to the financial support via the Ongoing Research Funding program (ORF‐2026‐754), King Saud University, Riyadh, Saudi Arabia, for funding this research.

## Conflicts of Interest

The authors declare no conflicts of interest.

## Supporting information

The supplementary data accompanying this article include the following: Figures S1–S15: Presenting the ^1^H, ^13^C, or DEPT NMR data for the new tetrazole derivatives **H**, **(1c‐2c/1d‐2d)** and **(1j‐2j)** & check cif, and CCDC reference: 2531191 for **F**, 2531190 for **G**, and 2531188 for 1j, containing the supplementary crystallographic data for this paper. These data can be obtained free of charge via http://www.ccdc.cam.ac.uk/conts/retrieving.html.

## Data Availability

The data that support the findings of this study are available in the supplementary material of this article.
