# Supplementary material for: Straightforward Synthesis, Spectroscopic Characterizations, Molecular Docking, Molecular Dynamics Simulations, and Comprehensive DFT Calculations of Novel Tetrazole 1,5 and 2,5‐Disubstituted Tetrazole Scaffolds
Source: ChemistryOpen. 2026 Aug 5;15(8):e70268. doi: 10.1002/open.70268 (PMC13439986; doi:10.1002/open.70268)
Supplement: Supplementary file 1 [file OPEN-15-e70268-s001.pdf]

Supporting information

Straightforward synthesis, spectroscopic characterizations, molecular docking, molecular dynamics simulations and comprehensive DFT calculations of novel tetrazole 1,5 and 2,5-disubstituted tetrazole scaffolds

Riham Sghyar[1,2], Ahmed Chelouan[1], Brahim El Ibrahimi [3], Olivier Blacque[4], Basavarajaiah Suliphuldevara Mathada[5], Prashantha Karunakar[6], Oussama Moussaoui[7], Mouad Lahyaoui[2], Joel T. Mague[8], Ali Aghmiz[1], Manal El-Gendy[9], Mohamed Hefnawy[9], Nada Kheira Sebbar[10*]

[1] Applied Chemistry, Microbiology and Biotechnologies, Department of Chemistry, Faculty of Sciences, University Abdelmalek Essaadi, Tetouan B.P. 2117, Morocco;
[2] Laboratory of Applied Organic Chemistry, Faculty of Science and Techniques, Sidi Mohamed Ben Abdellah University, B.P. 2202, Route Imouzzer, Fez 30050, Morocco.
[3] Department of Applied Chemistry, Faculty of Applied Sciences, 86153 Aït Melloul, Ibnou Zohr University, Morocco.
[4] University of Zurich, Department of Chemistry, winterthurerstrasse 190, CH-8057 Zurich
[5] Department of Chemistry, Dayananda Sagar Academy of Technology & Management (DSATM), Kanakapura Road, Udayapura, Bangalore- 560 082, Karnataka, INDIA
[6] Department of Biotechnology, Dayananda Sagar College of Engineering (Affiliated to Visvesvaraya Technological University, Belagavi), Bangalore, Karnataka, India.
[7] Laboratory of Organic Synthesis, Extraction and Valorization- Department of Chemistry, Faculty of Sciences Ain Chock, Hassan II University of Casablanca, BP:5366, Casablanca, Morocco;
[8] Department of Chemistry, Tulane University, New Orleans, LA 70118, USA.
[9] Department of Pharmaceutical Chemistry, College of Pharmacy, King Saud University, Riyadh, 11451, Saudi Arabia
[10] Laboratory of Heterocyclic Organic Chemistry, Drug Science Research Center, Pharmacochemistry Competence Center, Mohammed V University in Rabat, Faculty of Sciences, Av. Ibn Battouta, BP 1014 Rabat, Morocco;

***Corresponding authors**:* n.sebbar@uiz.ac.ma (N.K.SEBBAR).

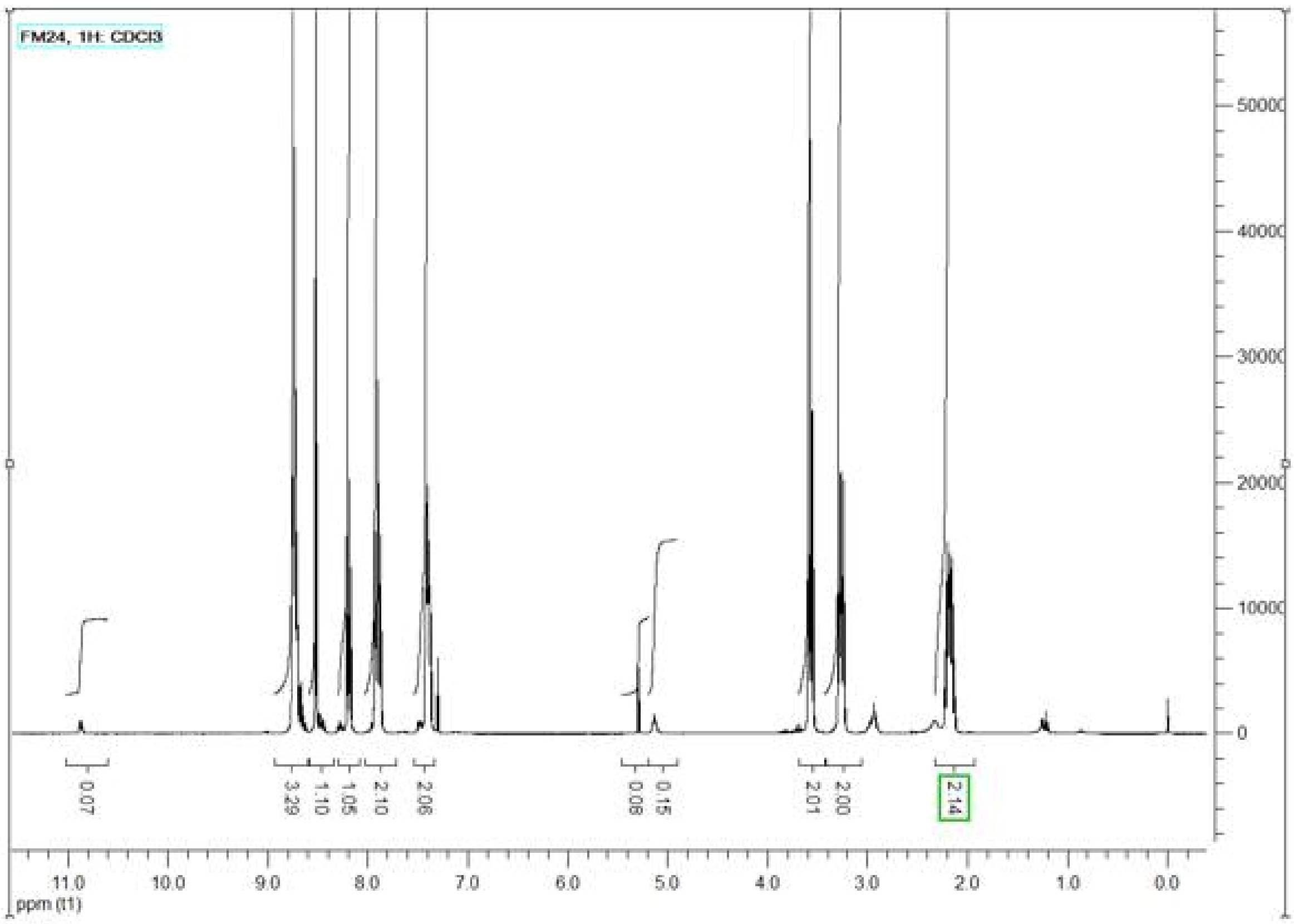

Figure S1: ^{1}H NMR spectrum ($CDCl_3$) of compound H: 4-(3-chloropropyl)-3,6-di(pyridin-2-yl)pyridazine

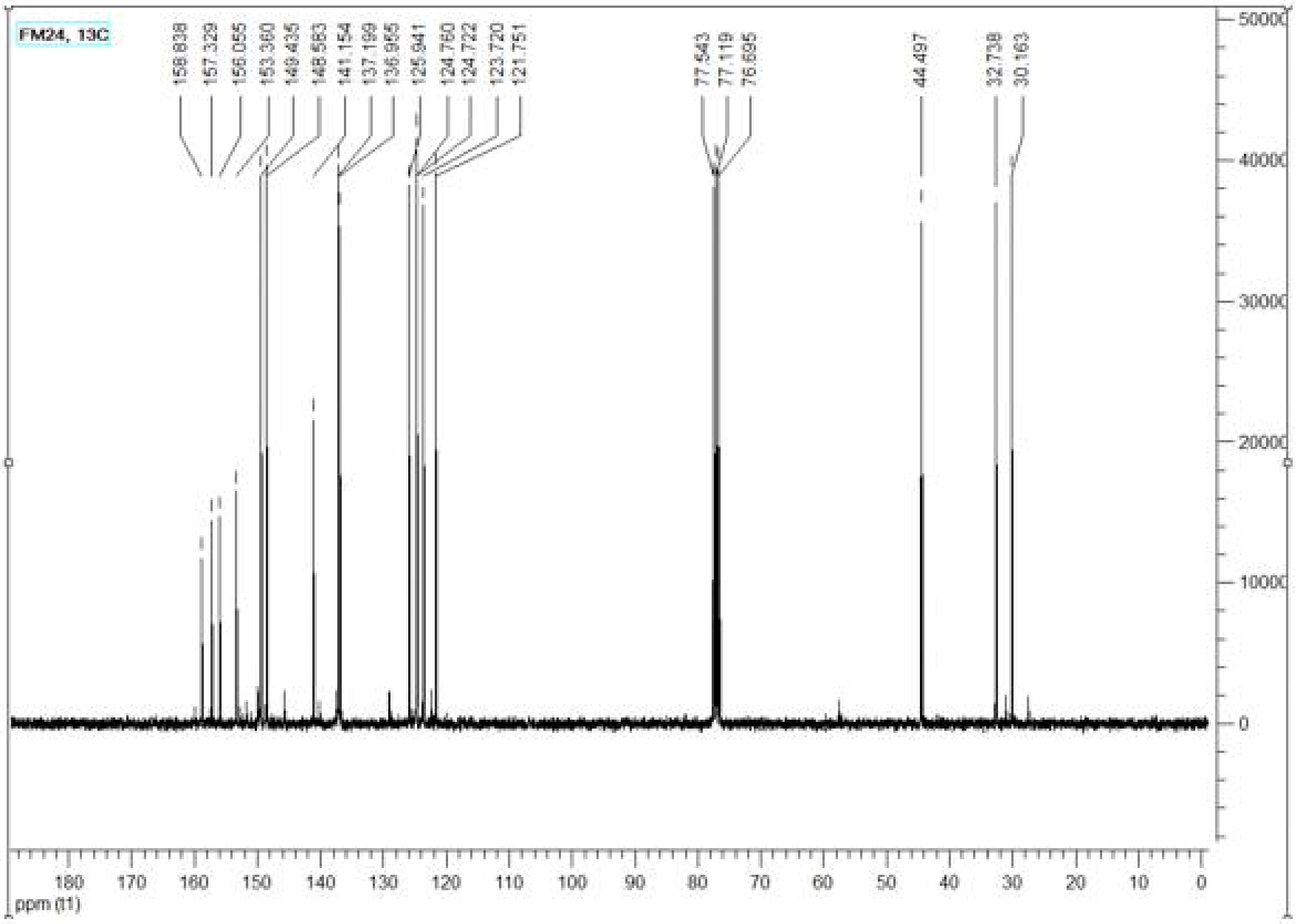

Figure S2: ^{13}C NMR spectrum ($CDCl_3$) of 4-(3-chloropropyl)-3,6-di(pyridin-2-yl)pyridazine

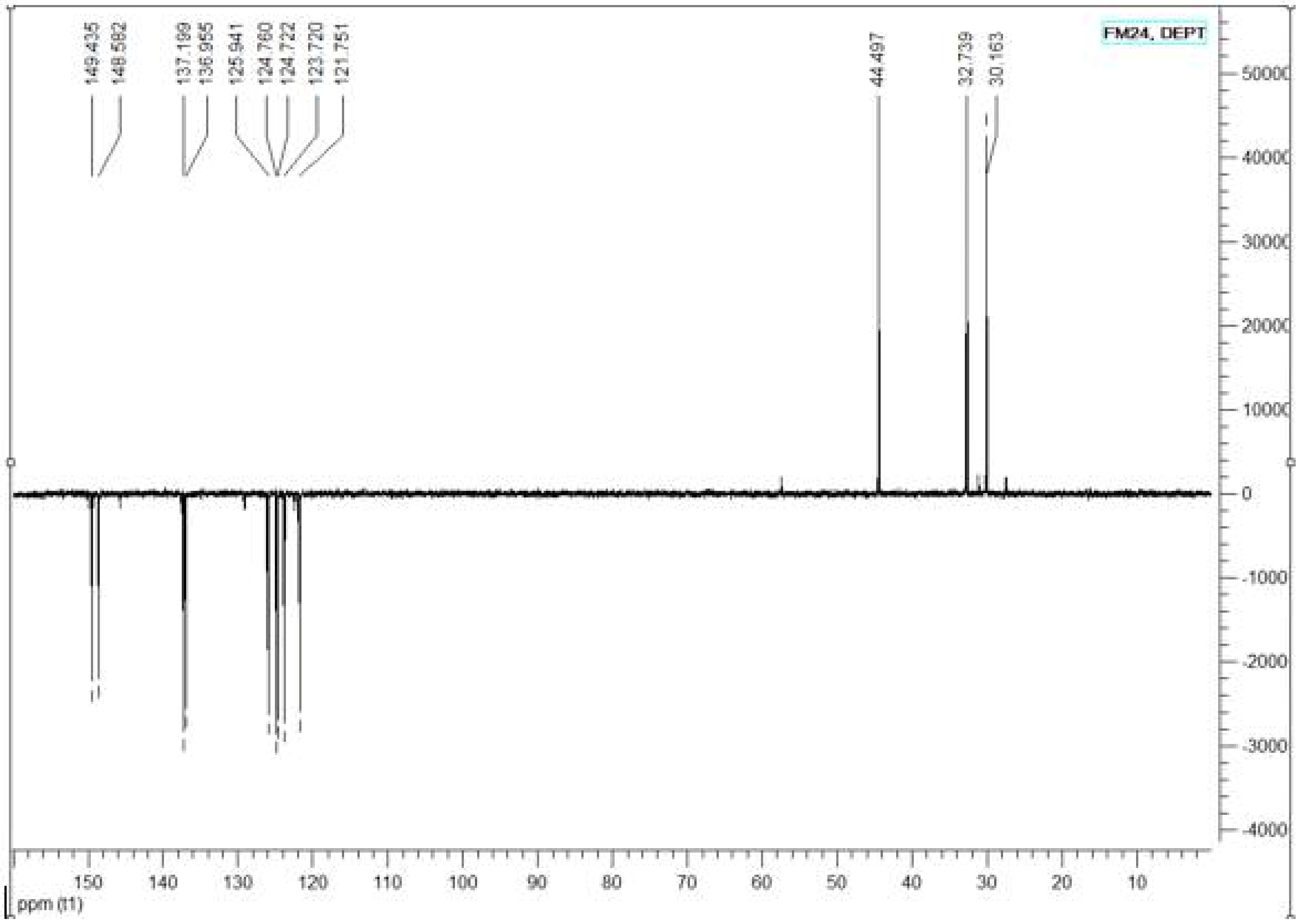

Figure S3: DEPT spectrum ($CDCl_3$) of 4-(3-chloropropyl)-3,6-di(pyridin-2-yl)pyridazine

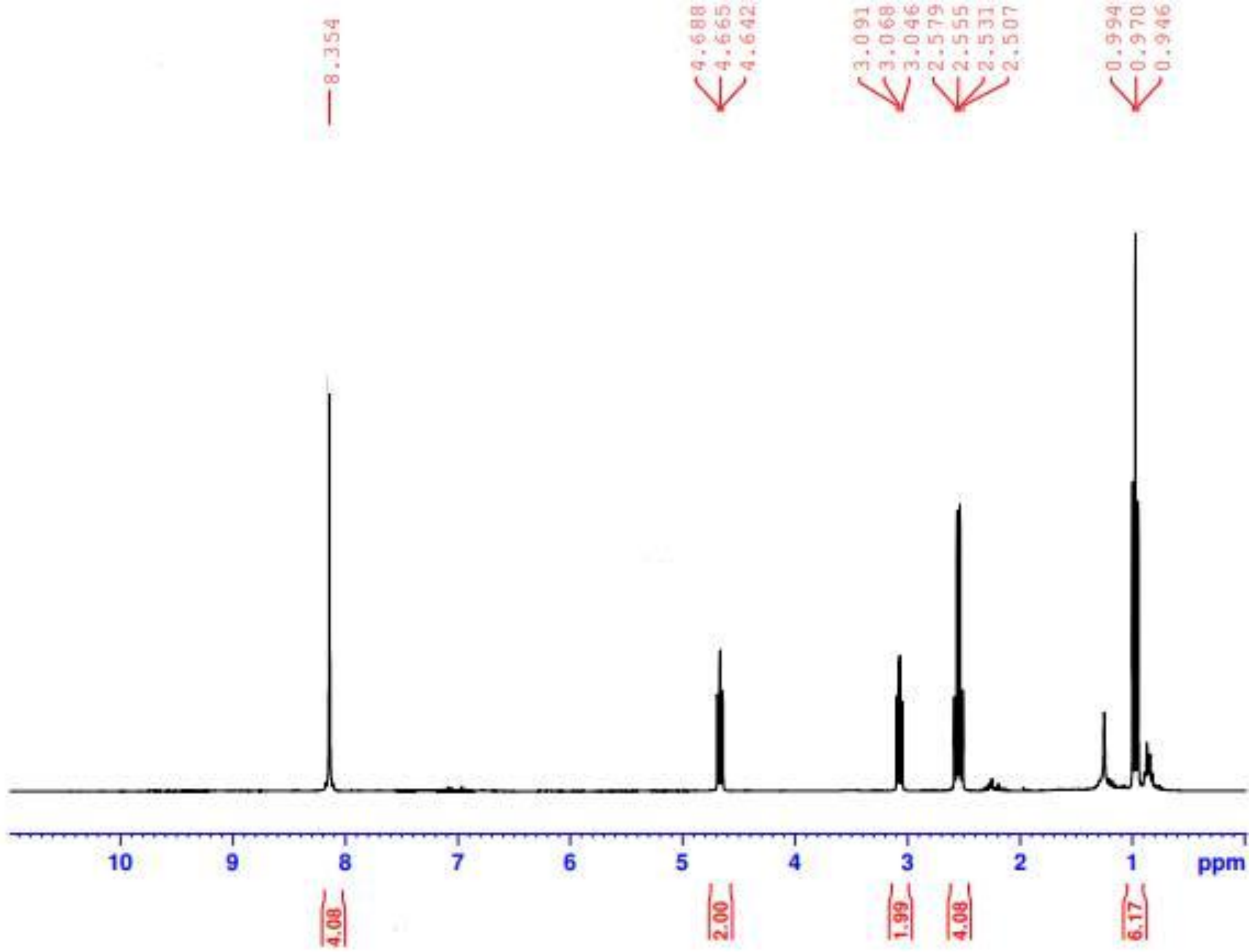

Figure S4: ^{1}H NMR spectrum ($CDCl_3$) of 1-(2-(5-(furan-2-yl)-2H-tetrazol-2-yl)ethyl)piperidine

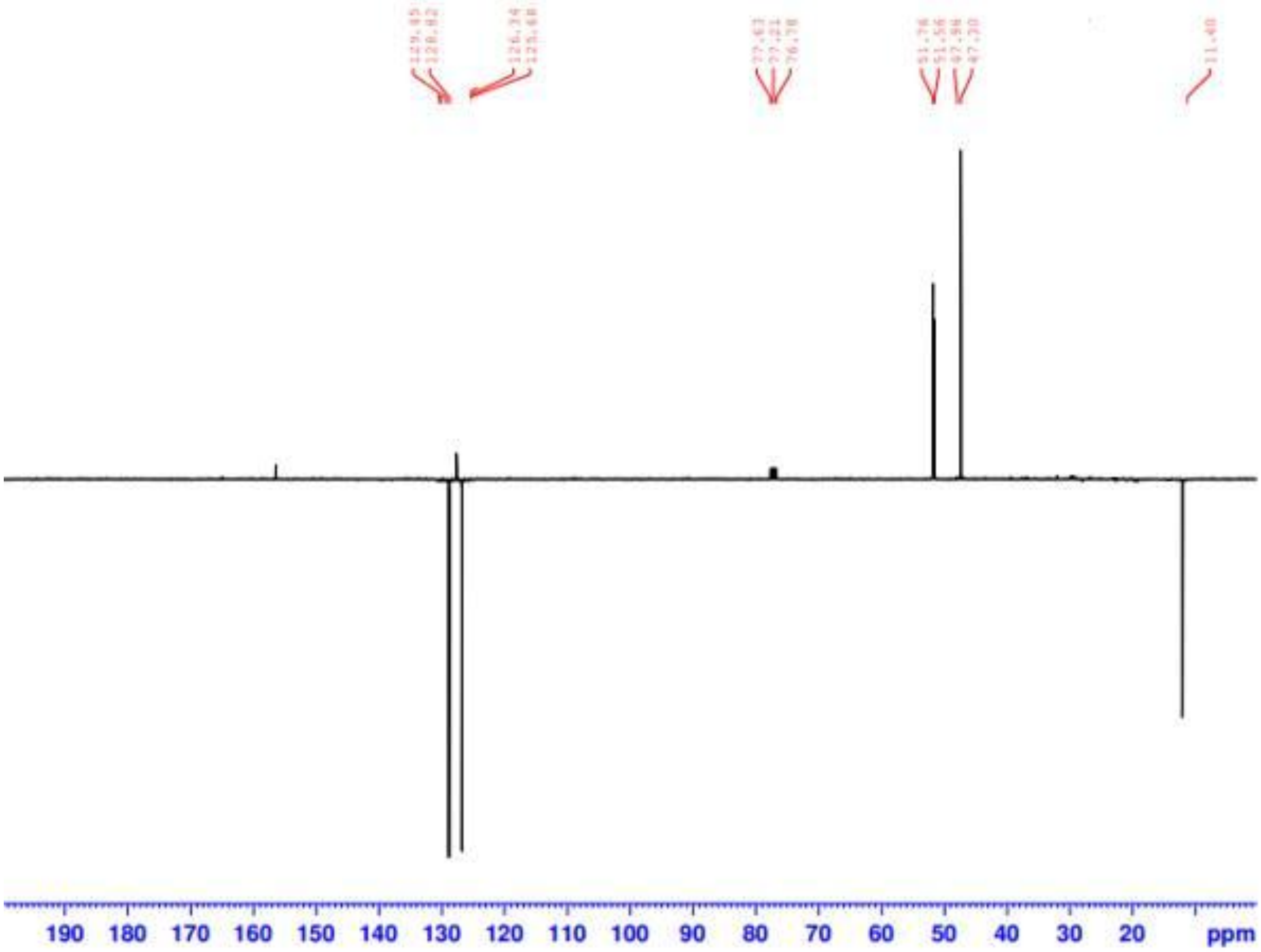

Figure S5: ^{13}C NMR spectrum ($CDCL_3$) of 1-(2-(5-(furan-2-yl)-2H-tetrazol-2-yl)ethyl)piperidine

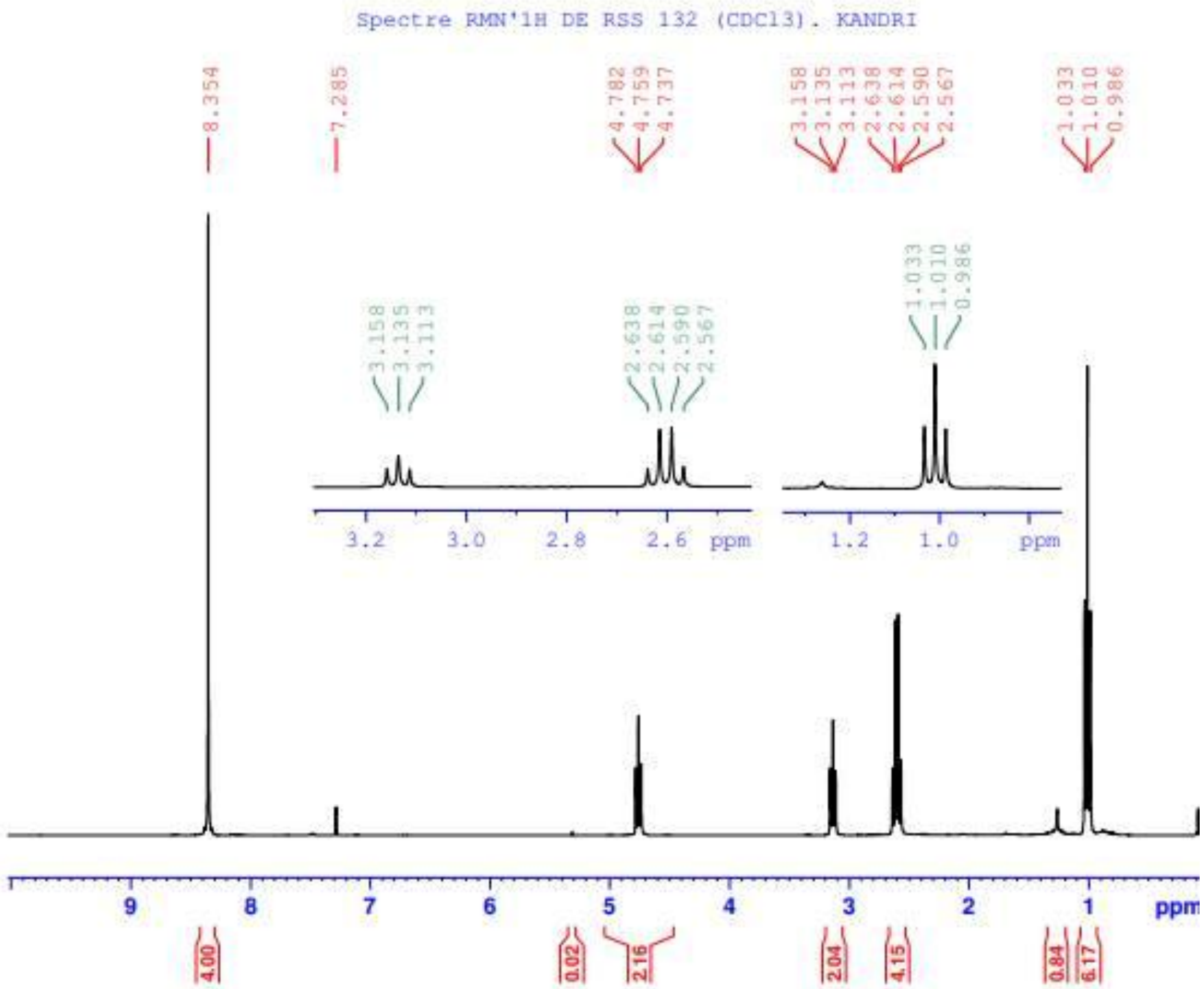

Figure S6: ^{1}H NMR spectrum ($CDCl_3$) of 1-(2-(5-(furan-2-yl)-2H-tetrazol-2-yl)ethyl)piperidine

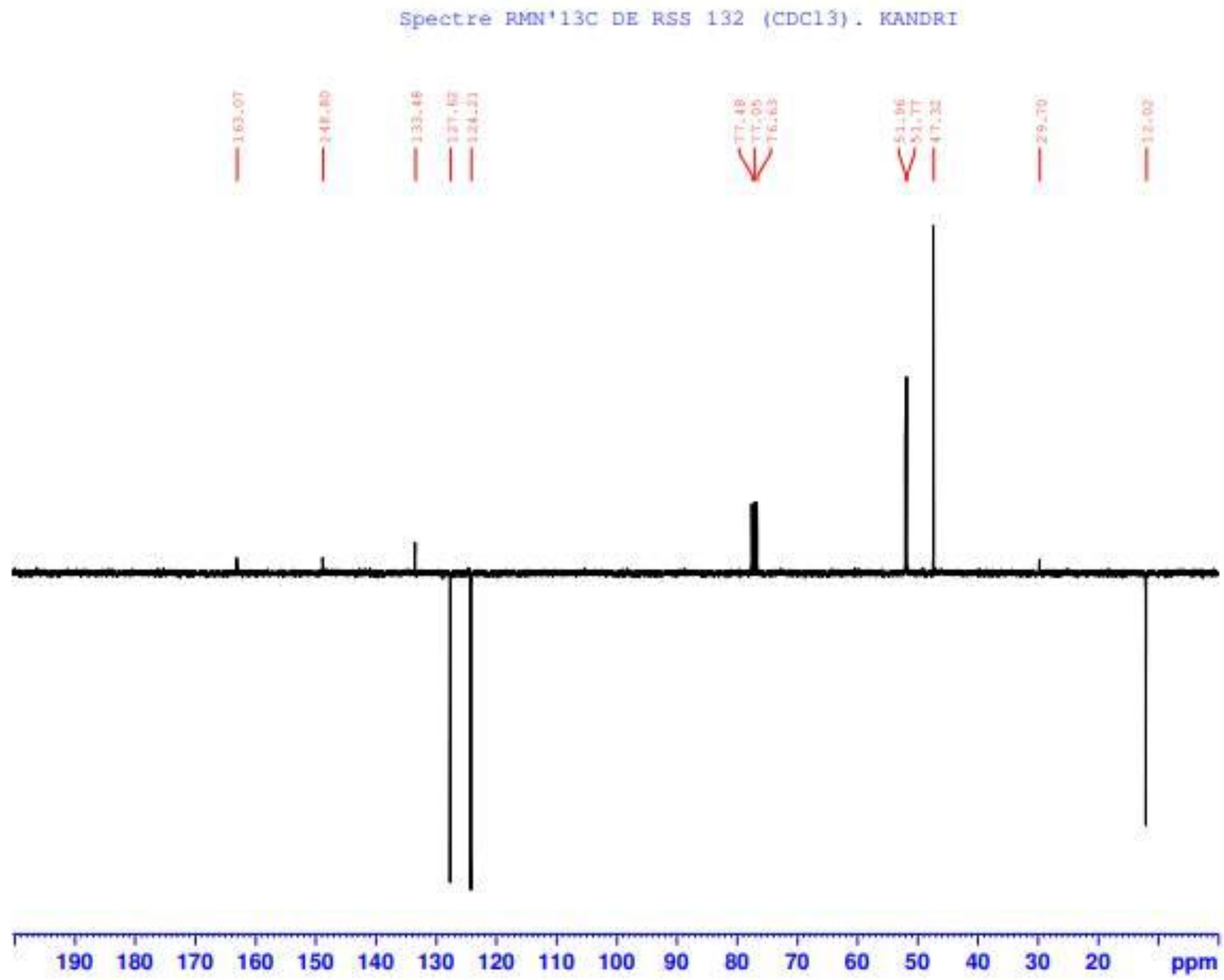

Figure S7: ^{13}C NMR spectrum ($CDCL_3$) of 1-(2-(5-(furan-2-yl)-2H-tetrazol-2-yl)ethyl)piperidine

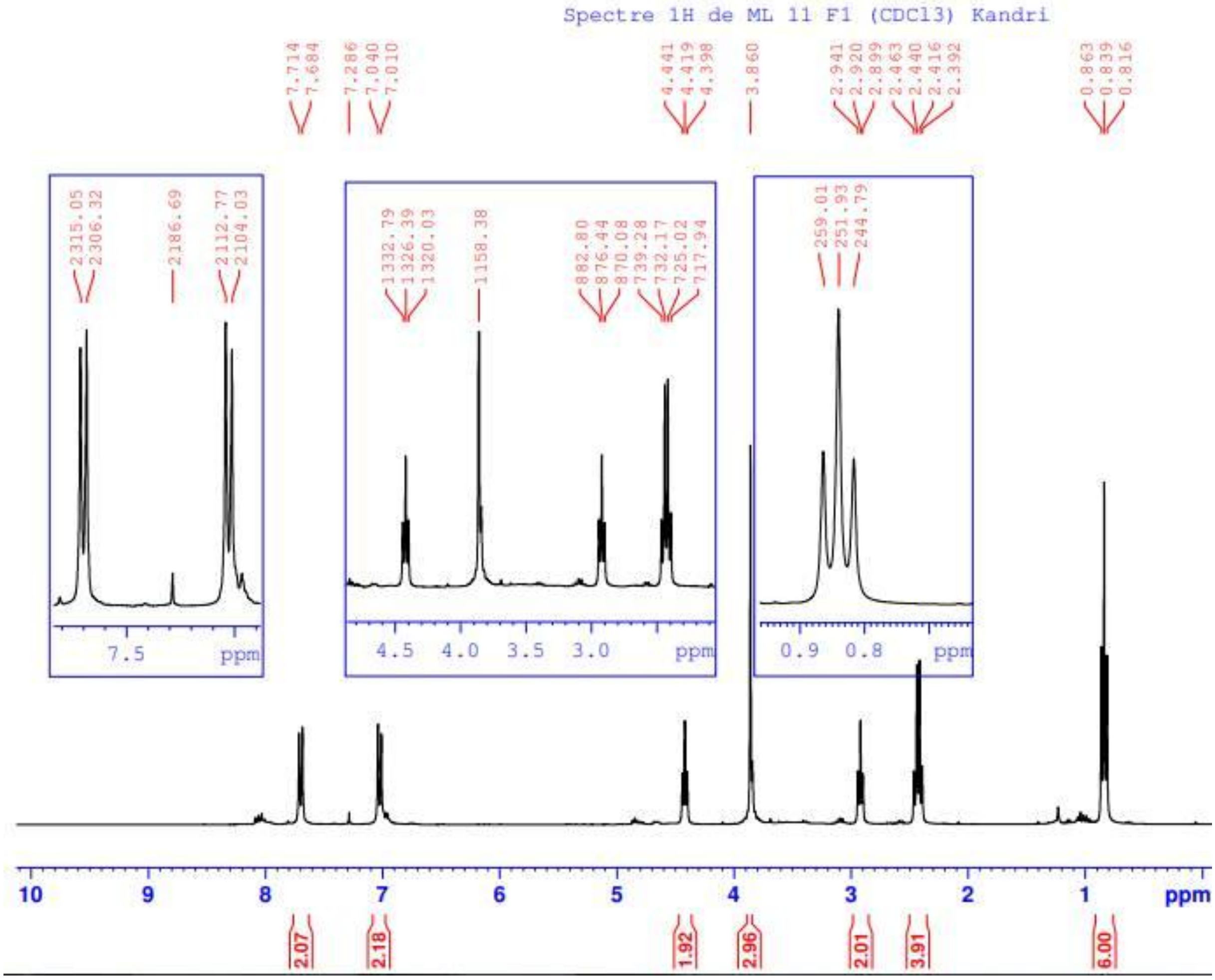

Figure S8: ^{1}H NMR spectrum ($CDCl_3$) of N,N-diethyl-2-(5-(4-methoxyphenyl)-1H-tetrazol-1-yl)ethanamine

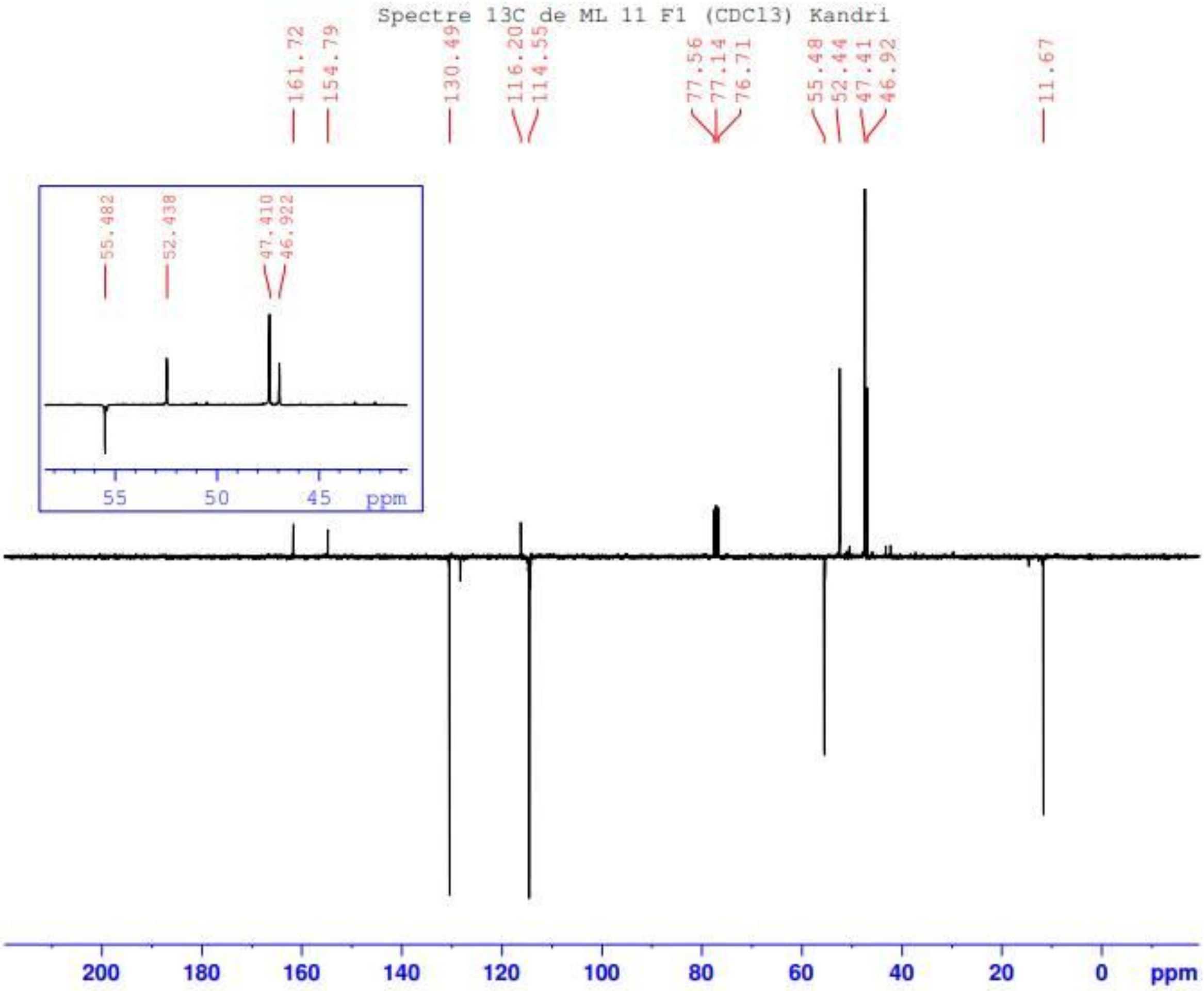

Figure S9: ^{13}C NMR spectrum ($CDCl_3$) of N,N-diethyl-2-(5-(4-methoxyphenyl)-1H-tetrazol-1-yl)ethanamine

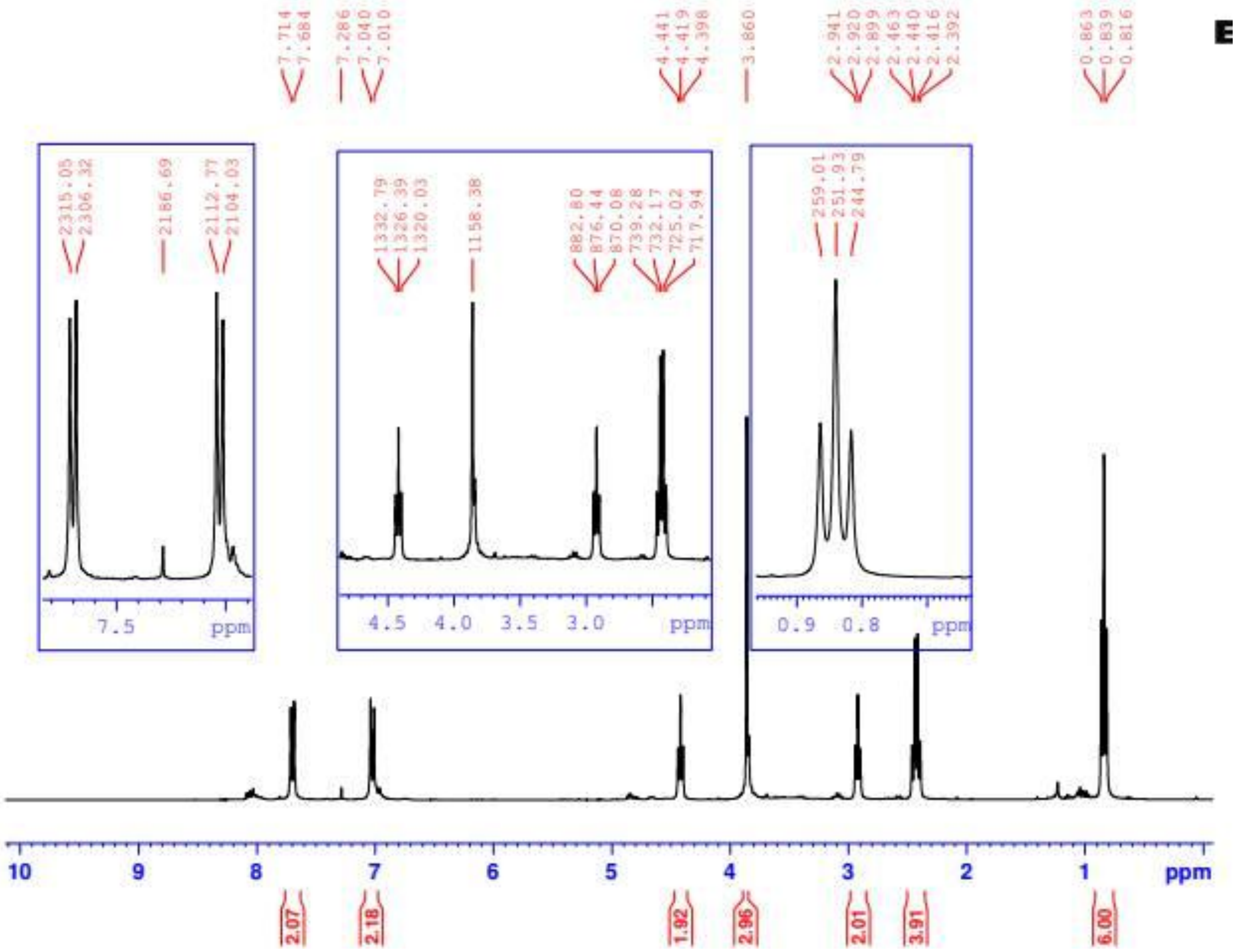

Figure S10: ^{1}H NMR spectrum ($CDCl_3$) of N,N-diethyl-2-(5-(4-methoxyphenyl)-2H-tetrazol-2-yl)ethanamine

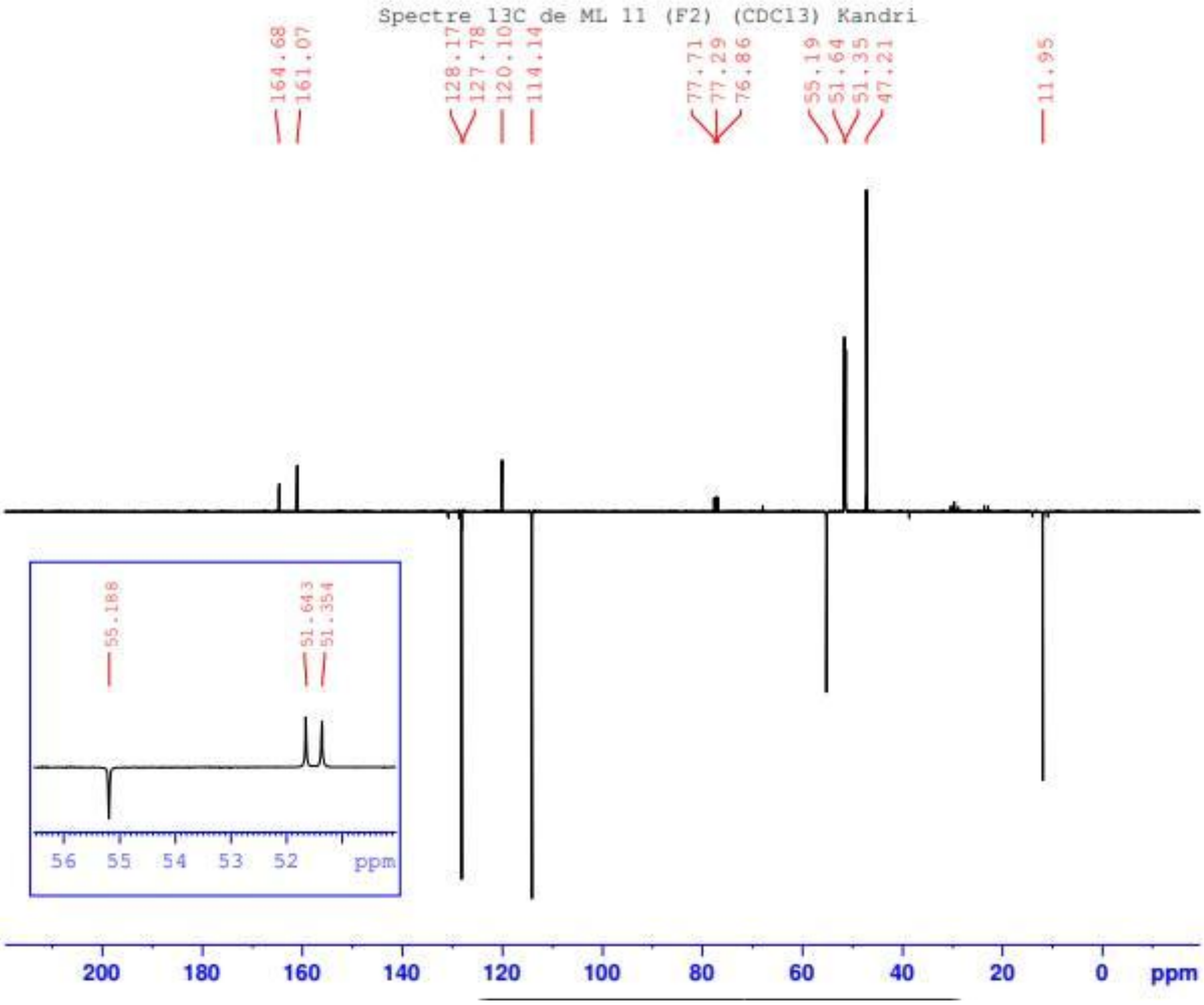

Figure S11: ^{13}C NMR spectrum ($CDCl_3$) of N,N-diethyl-2-(5-(4-methoxyphenyl)-2H-tetrazol-2-yl)ethanamine

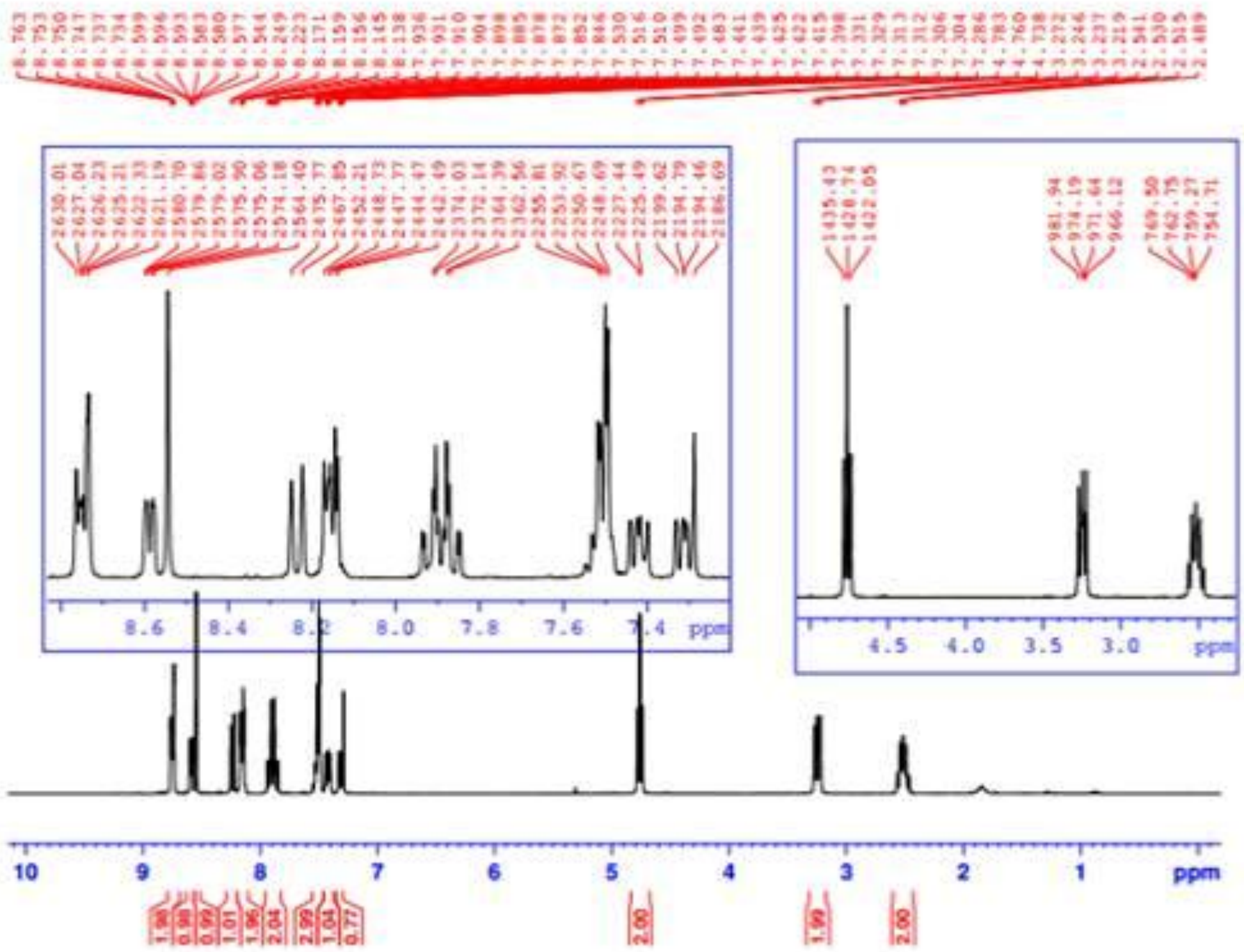

Figure S12: ^{1}H NMR spectrum ($CDCl_3$) of 4-(3-(5-phenyl-2H-tetrazol-2-yl)propyl)-3,6-di(pyridin-2-yl)pyridazine

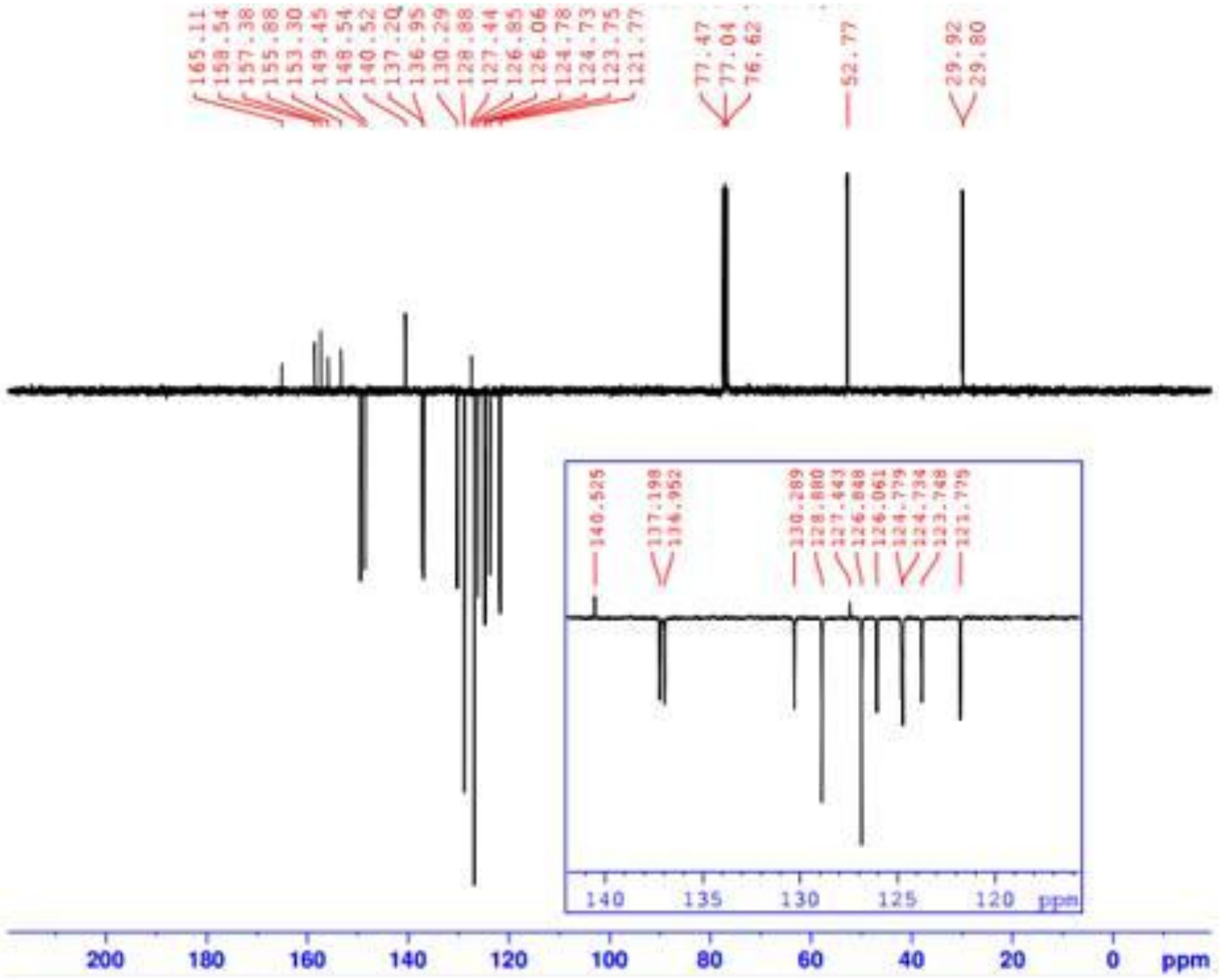

Figure S13: ^{13}C NMR spectrum ($CDCl_3$) of 4-(3-(5-phenyl-2H-tetrazol-2-yl)propyl)-3,6-di(pyridin-2-yl)pyridazine

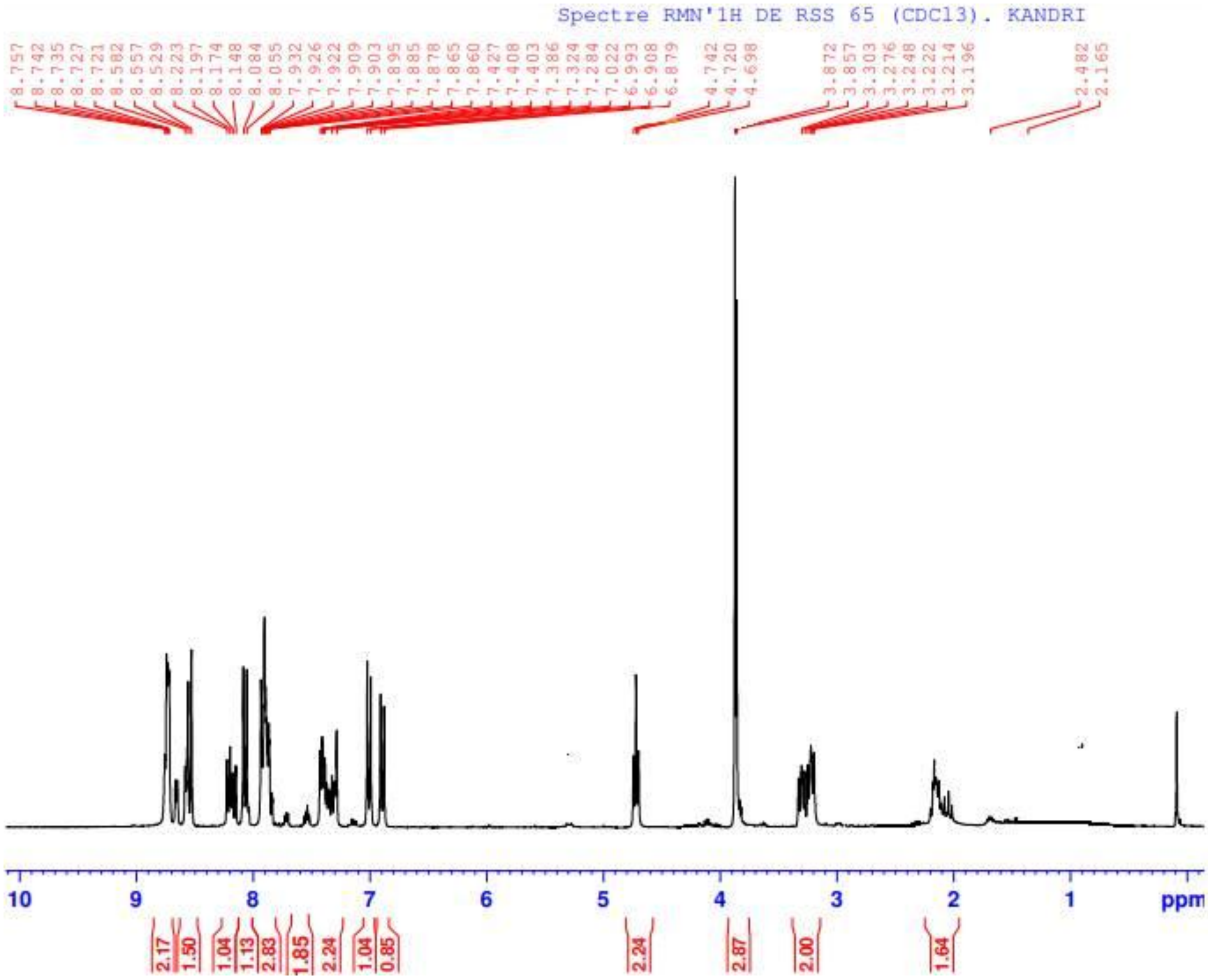

Figure S14: ^{1}H NMR spectrum ($CDCl_3$) of 4-(3-(5-phenyl-2H-tetrazol-2-yl)propyl)-3,6-di(pyridin-2-yl)pyridazine

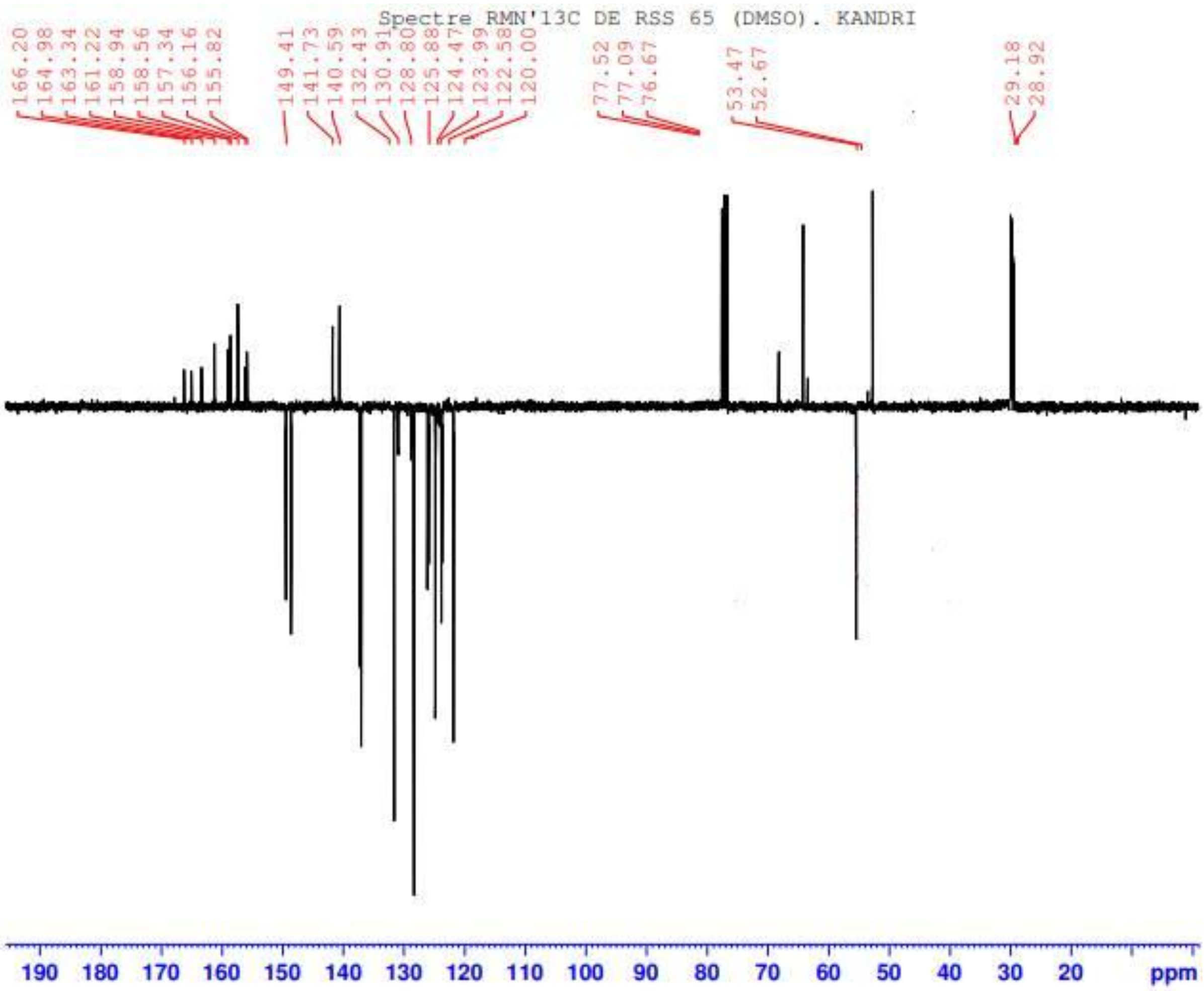

Figure S15: ^{13}C NMR spectrum ($CDCl_3$) of 4-(3-(5-phenyl-2H-tetrazol-2-yl)propyl)-3,6-di(pyridin-2-yl)pyridazine
